# Risk assessment of dietary factors in global pattern of ischemic heart disease mortality and disability-adjusted life years over 30 years

**DOI:** 10.3389/fnut.2023.1151445

**Published:** 2023-06-14

**Authors:** Fang Wang, Sumaira Mubarik, Yu Zhang, Wenqi Shi, Chuanhua Yu

**Affiliations:** ^1^Department of Biostatistics, School of Public Health, Xuzhou Medical University, Xuzhou, China; ^2^Center for Medical Statistics and Data Analysis, Xuzhou Medical University, Xuzhou, China; ^3^Key Laboratory of Human Genetics and Environmental Medicine, Xuzhou Medical University, Xuzhou, China; ^4^Department of Epidemiology and Biostatistics, School of Public Health, Wuhan University, Wuhan, China; ^5^School of Medicine, Hubei Polytechnic University, Huangshi, China; ^6^Global Health Institute, Wuhan University, Wuhan, China

**Keywords:** ischemic heart disease, global burden, death, dietary, temporal trends

## Abstract

**Objectives:**

The aim of this study was to investigate differences in the burden of ischemic heart disease (IHD)-related mortality and disability-adjusted life years (DALYs) caused by dietary factors, as well as the influencing factors with age, period, and cohort effects, in regions with different social-demographic status from 1990 to 2019.

**Methods:**

We extracted data on IHD mortality, DALYs, and age-standardized rates (ASRs) related to dietary risks from 1990 to 2019 as IHD burden measures. Hierarchical age–period–cohort analysis was used to analyze age- and time-related trends and the interaction between different dietary factors on the risk of IHD mortality and DALYs.

**Results:**

Globally, there were 9.2 million IHD deaths and 182 million DALYs in 2019. Both the ASRs of death and DALYs declined from 1990 to 2019 (percentage change: −30.8% and −28.6%, respectively), particularly in high and high-middle socio-demographic index (SDI) areas. Low-whole-grain, low-legume, and high-sodium diets were the three main dietary factors that increased the risk of IHD burden. Advanced age [RR (95%CI): 1.33 (1.27, 1.39)] and being male [1.11 (1.06, 1.16)] were independent risk factors for IHD mortality worldwide and in all SDI regions. After controlling for age effects, IHD risk showed a negative period effect overall. Poor diets were positively associated with increased risk of death but were not yet statistically significant. Interactions between dietary factors and advanced age were observed in all regions after adjusting for related variables. In people aged 55 and above, low intake of whole grains was associated with an increased risk of IHD death [1.28 (1.20, 1.36)]. DALY risks showed a similar but more obvious trend.

**Conclusion:**

IHD burden remains high, with significant regional variations. The high IHD burden could be attributed to advanced age, sex (male), and dietary risk factors. Dietary habits in different SDI regions may have varying effects on the global burden of IHD. In areas with lower SDI, it is recommended to pay more attention to dietary problems, particularly in the elderly, and to consider how to improve dietary patterns in order to reduce modifiable risk factors.

## Introduction

Ischemic heart disease (IHD), which is the leading cause of death globally, also accounts for the highest loss of disability-adjusted life years (DALYs). Income inequality, demographic changes, environmental risks, and unhealthy lifestyles pose challenges to the prevention and control of IHD in different regions, and it is expected that IHD will continue to pose a significant health threat to populations and societies (1, 2).

Research has indicated that metabolic risk factors are the main drivers of cardiovascular and cerebrovascular diseases (3). Metabolic abnormalities, however, can be largely attributed to modifiable risk factors such as poor lifestyle behaviors and unhealthy dietary habits (4, 5). For example, a high-sugar diet can lead to the increase of human uric acid level, impaired glucose tolerance, and changes in platelet function, thereby increasing the risk of coronary heart disease (6). Dietary risks were the most important level 2 risk factor leading to the highest IHD mortality and DALY rates in the Global Burden of Disease study (GBD) estimation (7). Among the 15 dietary risk factors involved in the GBD study, 87% were related to IHD, confirming the close relationship between diet and IHD (8).

In recent years, the Mediterranean diet, which focuses on olive oil, vegetables, fruits, nuts, and seeds, has been praised as the most comprehensive dietary pattern and has swept the world (9). This pattern promotes healthy metabolism and helps prevent cardiovascular disease (CVD) (9–11). Nevertheless, the high burden of IHD in the population suggests that this general awareness of health behaviors may not be effective in changing those behaviors in different regions or that there are regional differences in the transition from knowledge to action (12). Therefore, the current status of diet-related disease burden of IHD in different areas and its pattern changes across generations need to be further studied. The purpose of this study was to explore the differences in burden trends of IHD mortality and DALYs caused by dietary factors in different regions from 1990 to 2019, as well as the influencing factors such as age, period, and cohort effects.

## Materials and methods

### Data source

We collected and used the attributable burden of IHD data from the GBD 2019 study, obtained through the Global Health Data Exchange (GHDx) query tool,^1^ which is an ongoing global collaboration that uses all the latest available epidemiological data sources and improved standardized methodologies (13). GBD 2019 provides location, year, age, and sex estimates of 369 diseases and injuries and 87 risk factors in 204 countries and territories over the past 30 years (14). The input sources details and estimation methods used in GBD 2019 have been described elsewhere, and the data quality is globally recognized (1).

As a secondary study, we extracted estimates for deaths, DALYs, mortality, DALY rates, age-standardized death rates (ASDRs), age-standardized DALY rates, and their 95% uncertainty intervals (UIs) as primary measures of IHD burden. Data were collected from 1990 to 2019 by gender, selected dietary risk factors, and regions. ASRs were age-standardized using the GBD standard and reported per 100,000 population.

### Definition of disease and regions

IHD was defined based on the WHO clinical criteria and the International Statistical Classification of Diseases (GBD cause code B.2.2; ICD-10 codes I20-I25.9; ICD-9 codes 410–414.9). The socio-demographic index (SDI) (15) is calculated based on lag distributed income *per capita*, average educational attainment over the age of 15, and total fertility rate under 25 in order to evaluate the social development level comprehensively. It is used to position countries on the development continuum and to categorize the countries into five regions (high, high-medium, medium, low-medium, and low levels). We used this indicator as the basis for regional grouping.

### Identify risk factors

In GBD 2019, risks were organized into five hierarchical levels (7). Among the second-level risks in the GBD estimation system, the dietary factor was the leading cause of IHD deaths and DALYs. Therefore, we selected the top five dietary factors at the most detailed level 4—low whole grains (WG), low legumes, high sodium, high trans fatty acids (TFA), and low nuts and seeds (NS)—to generate attributable risk factors for IHD burden.

### Outcome variables

According to the comparative risk assessment (CRA), assuming that exposure levels of other risk factors remain unchanged, the theoretical minimum risk exposure distributions of various dietary risks were compared with the exposure distributions of a specific population. Next, the population attributable fractions (PAF) of each dietary risk were estimated (7), that is, the proportion of the IHD burden due to different dietary factors in the total IHD burden in a certain population. Based on the definition of PAF and the exposure distribution and exposure risk estimates at different exposure levels, the IHD deaths and DALYs attributable to dietary factors in different populations were estimated as:


$$PAF=\frac{\sum_{i}^{n}p_{i}\left(RR_{i}-1\right)}{\sum_{i}^{n}p_{i}\left(RR_{i}-1\right)+1}$$

where *p_i_* is the percentage of the population exposed to level *i* of risks, *n* is the total number of exposure level, and *RR_i_* is the relative risk at level *i*.

IHD deaths attributable to dietary factors were calculated by multiplying the PAFs and total disease-specific deaths. DALYs were calculated as the sum of years of life lost (YLL) and years lived with disability (YLD) (8). YLLs for IHD were calculated by subtracting the age at death from the life expectancy for a person of that age. YLDs were determined by multiplying the prevalence of each sequela by its disability weight. For DALYs attributed to dietary factors, YLL and YLD for IHD were multiplied by the corresponding PAF for each dietary factor. This resulted in the calculated attributable YLL and YLD, which were then added together to obtain the total DALYs caused by a certain dietary factor for IHD. We therefore obtained the absolute number and ASRs of deaths and DALYs for IHD due to dietary risks globally and in different SDI regions.

### Statistical analysis

#### Basic information

Descriptive analysis was used to characterize the burden of IHD worldwide and in various regions. The numbers of death cases, DALYs, and respective age-standardized rates (ASRs) in both genders combined over different years were compared. The trends in the ASRs reflect the alterations in disease patterns and risk factors. Additionally, the estimated annual percentage change (EAPC) was introduced to describe the ASR trend from 1990 to 2019.

#### Stratified association of risk with DALY and mortality

To measure the potential changes in IHD burdens associated with dietary factors in different SDI areas, we also described the mortality and DALYs of IHD stratified by different risk factors of age, period, and cohort. In the hierarchical age–period–cohort model (HAPC) framework, we analyzed the multivariate relationships between outcomes and three main sources for spatiotemporal variation: age, year, and cohort for each SDI region and globally. The patterns of IHD outcomes may be relevant to various socio-demographic and dietary risks and their ethnicity, using longitudinal panel evidence to indirectly assess the cohort effect. Since cohort effects can be calculated indirectly as a variation in time effects encountered by different age groups, we used the mixed-effects framework of hierarchical age–period–cohort to calculate the longitudinal panel data model (16).

Several mixed-effects models with fixed and random population-level effects and random slopes were used to explicitly assess differences in IHD mortality and DALYs among group levels over age (age effects) and time (period effects), in addition to, implicitly, the assessment period effect across populations of different age groups (cohort effects). Indeed, the mixed-effects model can make the three elements of age, period, and cohort effects nested so that they are no longer at the same level, which can solve the unrecognizable problem caused by collinearity problem in APC model (17, 18). In other words, the model can turn the period and cohort dimensions at the high-level into environmental variables and reflect their impact on the individual or population levels through the regression intercept and coefficient. Some individuals/population will be higher than the population mean (β_0_) at baseline, and some will be smaller. Some will have greater slopes than the population mean of β_1_, and some will be lower. This makes the model more flexible and versatile as it allows for heterogeneity of the baseline responses and responses over time. For IHD mortality analysis, the series of models were analyzed as follows:


(1) $$\begin{array}{l} DR_{it}=\left(\beta_{0}+b_{0i}\right)+\left(\beta_{1}+b_{1i}\right)Age_{it}+\left(\beta_{2}+b_{2i}\right)Year_{it}\\ \;\;\;\;\;\;\;\;\;\;\;\;+\left(\beta_{3}+b_{3i}\right)Gender_{i}+\left(\beta_{4}+b_{4i}\right)Risks_{it} \end{array}$$

(2) $$\begin{array}{c} DR_{it}=\left(\beta_{0}+b_{0i}\right)+\left(\beta_{1}+b_{1i}\right)Age_{it}+\left(\beta_{2}+b_{2i}\right)Year_{it}\\ +\left(\beta_{3}+b_{3i}\right)Gender_{i}+\left(\beta_{4}+b_{4i}\right)Risks_{it}\\ +\left(\beta_{5}+b_{5i}\right)Age_{it}\times Risks_{it}+\left(\beta_{6}+b_{6i}\right)\\ Gender_{i}\times Risks_{it}+\varepsilon_{it} \end{array}$$

(3) $$\begin{array}{c} DR_{it}=\left(\beta_{0}+b_{0i}\right)+\left(\beta_{1}+b_{1i}\right)Age_{it}+\left(\beta_{2}+b_{2i}\right)Year_{it}\\ +\left(\beta_{3}+b_{3i}\right)Gender_{i}+\left(\beta_{4}+b_{4i}\right)Risks_{it}\\ +\left(\beta_{5}+b_{5i}\right)Age_{it}\times Risks_{it}+\left(\beta_{6}+b_{6i}\right)\\ Gender_{i}\times Risks_{it}+\left(\beta_{7}+b_{7i}\right)Year_{it}\times Risks_{it}+\varepsilon_{it} \end{array}$$

(4) $$\begin{array}{c} DR_{it}=\left(\beta_{0}+b_{0i}\right)+\left(\beta_{1}+b_{1i}\right)Age_{it}+\left(\beta_{2}+b_{2i}\right)Year_{it}\\ +\left(\beta_{3}+b_{3i}\right)Gender_{i}+\left(\beta_{4}+b_{4i}\right)Risks_{it}\\ +\left(\beta_{5}+b_{5i}\right)Age_{it}\times Risks_{it}+\left(\beta_{6}+b_{6i}\right)\\ Gender_{i}\times Risks_{it}+\left(\beta_{7}+b_{7i}\right)Year_{it}\\ \times Risks_{it\left(\beta_{8}+b_{8i}\right)}Age_{it}\times Year_{it}+\varepsilon_{it} \end{array}$$

where *DR_it_* is the IHD mortality of population *i* at time *t*, *β*_0_,…, *β_k_* are the population mean intercept and slopes for related variables (fixed effects), *b*_0*i*_ is the difference between *β*_0_ and the intercept of *i* (random effect), *b*_1*i*_,…, *b_ki_*, are the differences between *β*_1_,…, *β_k_* and the slopes of *i* (random effects), and *ε_it_* is the random error within the population over time.

In the above series of models, the coefficient for age group can be interpreted as the overall age effect on IHD mortality after controlling for gender, year, and risk effects. The coefficient for year can be interpreted as the overall period effect after controlling for other variables. The coefficient of risks were the effects of different dietary risks on IHD mortality. The coefficients for age and risk interaction terms can be interpreted as differences in the experienced risk effect between different ages. The coefficients for gender and risk interaction terms can be interpreted as differences in the experienced risk effect between female (reference) and male people. The coefficients for the age and year interaction can be interpreted as differences in the experienced period effect across populations of varying ages (cohort effect). Overall, the exponential value of difference (coefficients) denoted the mortality/DALYs relative risk (RR) of a particular age group, period, and interactions terms relative to the reference groups. Similarly, DALYs were estimated using the same set of models. A likelihood ratio test was used to evaluate the existence of interaction effects. The degree of model fitting was evaluated by deviance, the Akaike information criterion (AIC), and Bayesian information criterion (BIC) to select the most appropriate model. After comparison, model 2 was finally selected to be included in the study.

#### Age, period, and cohort effects on IHD burden by major dietary risks

We additionally analyzed the age, period, and cohort effects on IHD burden (mortality and DALYs) attributable to the top three dietary risks of diet low in whole grains, diet low in legumes, and diet high in sodium in different SDI regions by using an APC model based on intrinsic estimation (IE) algorithm (19):


$$Y=\log\left(M\right)=\mu +\alpha Age_{i}+\beta Period_{j}+\gamma Cohort_{k}+\varepsilon_{it}$$

where *M* is defined as the mortality rates and DALYs. *α*, *β*, and *γ* refer to the coefficients of three dimensions, and *μ* and *ε* are defined as the intercept and random error. The standard error (SE) coefficient and risk ratios were calculated. The above statistical description and analyses were all performed using the R program (Version 4.1.2, R core team). Results with *p* < 0.05 were considered statistically significant.

## Results

### Overall trend in mortality and DALYs of ischemic heart disease

Globally, the number of death cases of IHD has increased by 60.43% from 5.70 million in 1990 to 9.14 million in 2019, and the number of DALYs increased by 50.35% from 121.07 million to 182.03 million (Table 1). The overall ASRs of death decreased in the same period (EAPC = −30.8% [95% UI: −34.83 to −27.17]) from 170.45 to 117.95 per 100,000 persons. In terms of SDI regions, the number of deaths and DALYs increased in all subregions except for high-SDI regions. In contrast, the ASDRs in all regions decreased, but the corresponding decrease was only significant in high- and high-middle-SDI areas. The variation trend of DALYs during this period was similar to that of ASDRs, and the decrease was not obvious in low- and low-middle-SDI regions.

**Table 1:** The death cases, DALYs, and related age-standardized rates (ASRs) of ischemic heart disease in 1990 and 2019 and its temporal trends from 1990 to 2019.

Types	1990		2019		1990–2019
	Cases [No. × 10^3^ (95% UI)]	ASR per 100,000 [No. (95% UI)]	Cases [No. × 10^3^ (95% UI)]	ASR per 100,000 [No. (95% UI)]	EAPC [No. (95% UI)]
Death
Overall	5,695.89 (5,405.19, 5,895.40)	170.45 (159.61, 176.94)	9,137.79 (8,395.68, 9,743.55)	117.95 (107.83, 125.92)	−30.8 (−34.83, −27.17)
Sex
Male	3,022.46 (2,895.63, 3,133.48)	205.24 (194.29, 213.05)	4,968.25 (4,591.34, 5,344.57)	144.6 (132.87, 154.96)	−29.55 (−34.72, −24.81)
Female	2,673.43 (2,478.70, 2,815.92)	141.73 (130.00, 149.72)	4,169.54 (3,680.48, 4,521.72)	95.07 (83.91, 103.11)	−32.92 (−37.96, −28.36)
SDI
High SDI	1,688.79 (1,572.96, 1,744.99)	162.39 (150.62, 168.15)	1,447.27 (1,270.02, 1,553.84)	67.1 (60.07, 71.54)	−58.68 (−60.30, −56.69)
High-middle SDI	1,870.95 (1,782.68, 1,923.52)	209.2 (196.25, 216.23)	2,658.29 (2,411.87, 2,832.48)	135.41 (122.68, 144.44)	−35.28 (−39.01, −31.69)
Middle SDI	1,151.13 (1,087.98, 1,217.99)	143.11 (133.18, 152.13)	2,824.55 (2,576.47, 3,047.02)	134.12 (121.51, 145.22)	−6.28 (−14.41, 1.84)
Low-middle SDI	712.69 (654.08, 773.46)	144.21 (132.05, 156.58)	1,646.06 (1,488.07, 1,801.77)	136.59 (122.96, 149.50)	−5.28 (−15.25, 5.09)
Low SDI	269.14 (240.68, 301.92)	139.20 (124.00, 156.79)	556.60 (495.17, 627.06)	127.99 (113.13, 143.90)	−8.05 (−20.59, 3.17)
DALYs
Overall	1,21,068.87 (1,16,357.40, 1,25,633.90)	3,143.28 (3,012.81, 3,257.17)	1,82,030.14 (1,70,206.78, 1,93,504.63)	2,243.54 (2,098.70, 2,385.01)	−28.62 (−24.16, −33.28)
Sex
Male	72,227.38 (69,305.13, 75,003.87)	4,003.52 (3,840.47, 4,147.26)	1,10,683.66 (1,02,235.95, 1,18,954.62)	2,899.51 (2,681.07, 3,117.87)	−27.58 (−33.40, −21.84)
Female	48,841.50 (46,134.15, 51,457.89)	2,366.31 (2,222.27, 2,491.26)	71,346.48 (64,769.33, 77,093.70)	1,637.86 (1,486.52, 1,769.83)	−30.78 (−36.85, −25.39)
SDI
High SDI	29,372.65 (28,133.32, 30,043.01)	2,833.48 (2,714.36, 2,898.17)	22,126.94 (20,524.86, 23,331.05)	1,188.04 (1,115.22, 1,246.49)	−58.07 (−59.46, −56.20)
High-middle SDI	37,654.49 (36,362.34, 38,712.49)	3,692.48 (3,539.46, 3,799.84)	46,960.74 (43,498.58, 49,829.05)	2,346.48 (2,172.97, 2,492.22)	−36.45 (−40.41, −32.47)
Middle SDI	28,068.28 (26,555.47, 29,665.87)	2,755.61 (2,603.65, 2,909.61)	59,649.47 (54,813.58, 64,435.73)	2,476.04 (2,273.77, 2,675.65)	−10.15 (−18.26, −2.17)
Low-middle SDI	18,861.31 (17,327.36, 20,460.10)	3,040.68 (2,796.16, 3,299.79)	39,251.98 (35,477.77, 43,178.51)	2,831.95 (2,564.04, 3,104.29)	−6.86 (−17.50, 4.31)
Low SDI	7,044.40 (6,312.59, 7,911.42)	2,908.58 (2,609.96, 3,254.17)	13,935.07 (12,423.47, 15,691.65)	2,606.43 (2,330.38, 2, 923.57)	−10.39 (−22.27, 1.36)

DALYs, disability-adjusted life years; ASR, age-standardized rate; UI, uncertain interval; EAPC, estimated annual percentage change; SDI, socio-demographic index.

### IHD mortality and DALYs attributable to dietary factors

As shown in Supplementary Figure S1, the ASDR attributable to high diet in TFA was highest in 1990 in high-SDI regions, but it declined the most, reaching the lowest point by 2019. Apart from high TFA intake, IHD mortality from all other four dietary factors was highest in high-middle-SDI regions in 1990 and remained high in 2019, despite a decline over 30 years. In addition, deaths attributed to five dietary factors in high-SDI regions all fell to their lowest levels in 2019.

**Figure 1:**
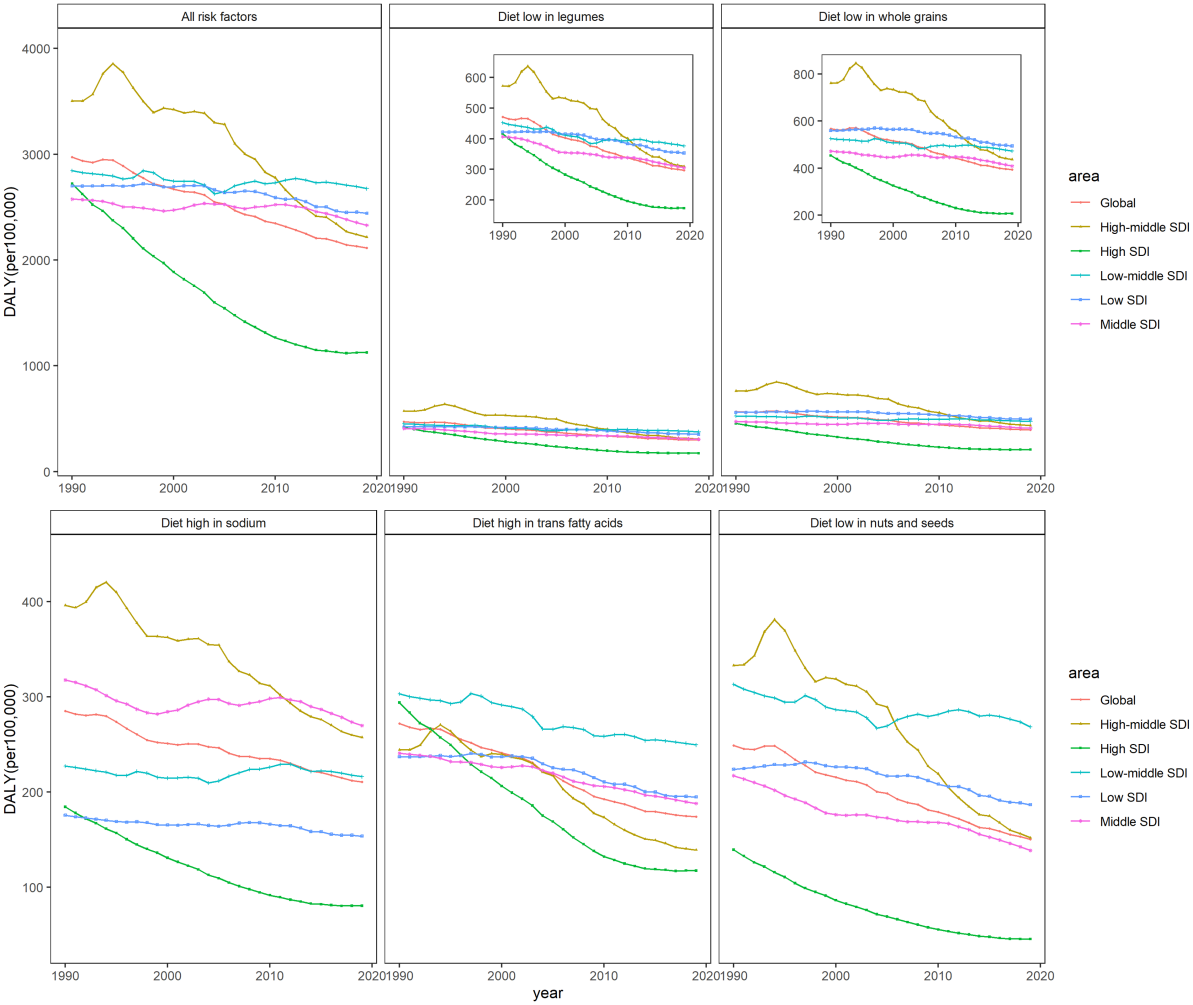
The age-standardized rates of DALYs attributable to dietary factors from 1990 to 2019 in world SDI regions and globally, both sexes.

The trends of IHD mortality caused by diets with low levels of legumes and whole grains were similar in all regions, being highest in 2019 in high-middle-SDI regions, as noted above. By 2019, IHD mortality attributable to high-sodium levels was higher in middle-SDI regions than in others. The attributable mortality of the diet high in TFA and low in nuts and seeds was at the highest level in low-middle-SDI regions in 2019. The attributed DALYs showed a similar trend (Figure 1), but the fluctuations were more obvious than that of mortality. DALY caused by low whole-grains intake was highest in areas with low SDI. This suggests that different dietary habits in different SDI regions may affect the burden of IHD populations worldwide to varying degrees.

### Age-, period-, and cohort-related trends and interaction with dietary risks

In terms of age, period, and their risk interaction effects, we observed a significant increase in the risk of death among age groups of 55 years and older compared with the younger populations, both globally and in all SDI regions, assuming the impact of other variables remained unchanged (Table 2). The area with the highest age-related risk of death was the high-middle-SDI region (RR = 1.50, 95%CI: 1.41, 1.60), followed by the middle-SDI region [RR (95%CI): 1.37 (1.33, 1.42)]. The risk of death in male people was higher than that of female people in all regions, and the RR (95%CI) in the high-middle-SDI area was the highest [1.18 (1.11, 1.26)].

**Table 2:** Mixed-effect model estimates (RR, 95% CIs) predicting ischemic heart disease (IHD) death risk, globally, and by SDI region separately.

Value		Global	High SDI	High-middle SDI	Middle SDI	Low-middle SDI	Low SDI
Age group (year)	
	10–54	1 (reference)	1 (reference)	1 (reference)	1 (reference)	1 (reference)	1 (reference)		≥55	1.33 (1.27, 1.39)	1.20 (1.13, 1.27)	1.50 (1.41, 1.60)	1.37 (1.33, 1.42)	1.25 (1.20, 1.29)	1.20 (1.17, 1.24)
Sex
	Female	1 (reference)	1 (reference)	1 (reference)	1 (reference)	1 (reference)	1 (reference)
	Male	1.11 (1.06, 1.16)	1.07 (1.01, 1.14)	1.18 (1.11, 1.26)	1.12 (1.08, 1.16)	1.08 (1.04, 1.12)	1.05 (1.02, 1.08)
Year
	1990	1 (reference)	1 (reference)	1 (reference)	1 (reference)	1 (reference)	1 (reference)
	1999	0.98 (0.95, 1.00)	0.95 (0.91, 0.99)	0.98 (0.95,1.02)	0.99 (0.97, 1.01)	0.99 (0.97, 1.01)	1.00 (0.98, 1.02)
	2009	0.95 (0.92, 0.98)	0.90 (0.86, 0.93)	0.94 (0.90, 0.97)	0.99 (0.97, 1.01)	0.99 (0.97, 1.01)	0.99 (0.97, 1.01)
	2019	0.93 (0.91, 0.96)	0.88 (0.85, 0.92)	0.90 (0.86, 0.93)	0.98 (0.96, 1.00)	0.99 (0.96, 1.01)	0.98 (0.96, 1.00)
Risks
	Diet high in sodium	1 (reference)	1 (reference)	1 (reference)	1 (reference)	1 (reference)	1 (reference)
	Diet high in TFA	1.03 (0.98, 1.09)	1.01 (0.94, 1.09)	1.07 (0.99, 1.15)	1.04 (1.00, 1.08)	1.01 (0.97, 1.06)	1.01 (0.97, 1.05)
	Diet low in legumes	1.02 (0.97, 1.08)	1.01 (0.93, 1.08)	1.05 (0.97, 1.14)	1.03 (0.99, 1.07)	1.01 (0.96, 1.05)	0.99 (0.96, 1.03)
	Diet low in nuts and seeds	1.03 (0.98, 1.09)	1.02 (0.95, 1.10)	1.06 (0.98, 1.15)	1.04 (1.00, 1.08)	1.01 (0.97, 1.06)	1.01 (0.97, 1.05)
	Diet low in whole grains	1.02 (0.96, 1.07)	1.00 (0.93, 1.08)	1.04 (0.96, 1.12)	1.02 (0.98, 1.06)	1.00 (0.95, 1.04)	0.98 (0.95, 1.02)
Risk age interaction	
	≥55 × Diet high in TFA	0.96 (0.90, 1.02)	1.09 (1.00, 1.19)	0.84 (0.77, 0.92)	0.90 (0.86, 0.95)	1.03 (0.98, 1.08)	1.02 (0.98, 1.07)
	≥55 × Diet low in legumes	1.15 (1.08, 1.22)	1.23 (1.13, 1.34)	1.15 (1.06, 1.26)	1.04 (0.99, 1.09)	1.16 (1.10, 1.21)	1.20 (1.15, 1.25)
	≥55 × Diet low in nuts and seeds	0.93 (0.87, 0.99)	0.95 (0.87, 1.04)	0.91 (0.83, 0.99)	0.87 (0.83, 0.91)	1.03 (0.98, 1.08)	1.00 (0.95, 1.04)
	≥55 × Diet low in whole grains	1.28 (1.20, 1.36)	1.28 (1.17, 1.40)	1.41 (1.29, 1.54)	1.14 (1.08, 1.19)	1.26 (1.20, 1.32)	1.37 (1.31, 1.43)
Risk sex interaction	
	Male×Diet high in TFA	0.94 (0.88, 1.00)	0.98 (0.90, 1.07)	0.87 (0.80, 0.96)	0.93 (0.88, 0.97)	0.98 (0.93, 1.03)	0.99 (0.95, 1.04)
	Male×Diet low in legumes	0.97 (0.91, 1.03)	1.01 (0.92, 1.10)	0.92 (0.84, 1.00)	0.96 (0.91, 1.00)	1.01 (0.96, 1.06)	1.03 (0.99, 1.08)
	Male×Diet low in nuts and seeds	0.93 (0.88, 0.99)	0.96 (0.88, 1.05)	0.88 (0.81, 0.96)	0.92 (0.88, 0.97)	0.99 (0.94, 1.04)	0.99 (0.95, 1.04)
	Male×Diet low in whole grains	0.99 (0.93, 1.05)	1.02 (0.94, 1.12)	0.95 (0.87, 1.04)	0.98 (0.93, 1.02)	1.03 (0.99, 1.09)	1.06 (1.01, 1.11)
AIC		450.01	494.23	497.17	418.60	422.36	409.01
BIC		497.66	541.87	544.81	466.24	470.00	456.65

SDI, Socio-demographic index; TFA, trans fatty acids; AIC, Akaike information criterion; BIC, Bayesian information criterion; CI, confidence intervals; RR, relative risk.

After controlling for age effects, there was a negative period effect on IHD mortality from 1990 to 2019. In high-SDI areas, the risk of death declined significantly over time, with a 12% reduction in 2019 compared with 1990. These correlation effects were not statistically significant in the middle- or lower-SDI regions. Globally, diet-related risks were positively associated with the increased risk of death, but the difference was not statistically significant. A significant interaction between risk and age was observed in the global population aged 55 and older, and it was associated with low intake of legumes and whole grains. The risk of death from IHD was increased in people aged 55 years and older who consumed low levels of whole grains (RR = 1.28, 95%CI: 1.20, 1.36). Low level of whole grains increased the risk of death from IHD among people aged 55 and above (RR = 1.28, 95% CI: 1.20, 1.36). As shown in Supplementary Table S1, a similar but more pronounced trend can be found in the risk analysis of DALYs.

### Comparison of estimated APC effects by SDI regions in major dietary factors

**Figure 2:**
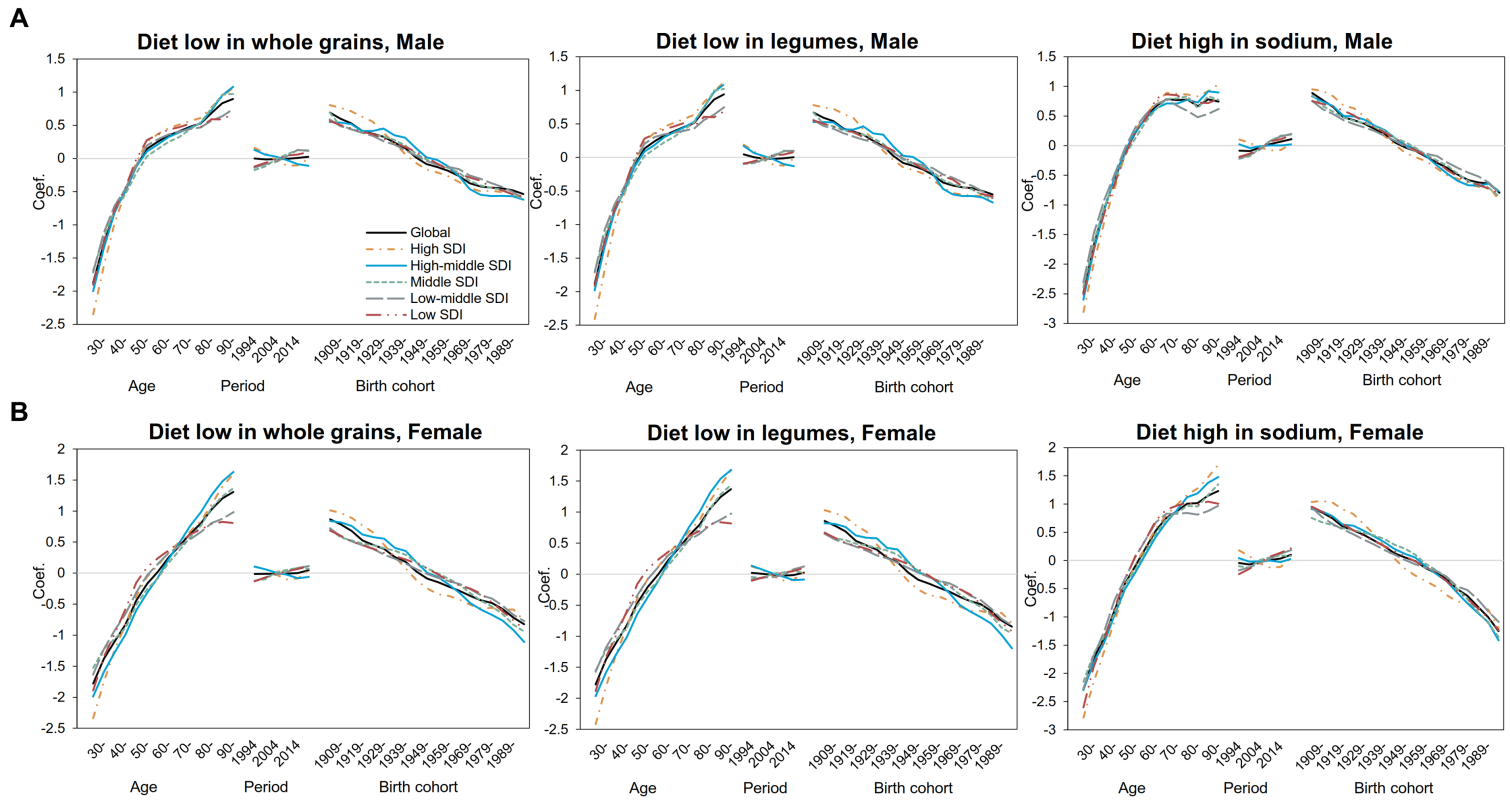
Age–period–cohort-related trends in IHD DALYs from 1990 to 2019 globally and SDI quintiles attributable to most common dietary risks stratified by sex **(A)** Male **(B)** Female.

**Figure 3:**
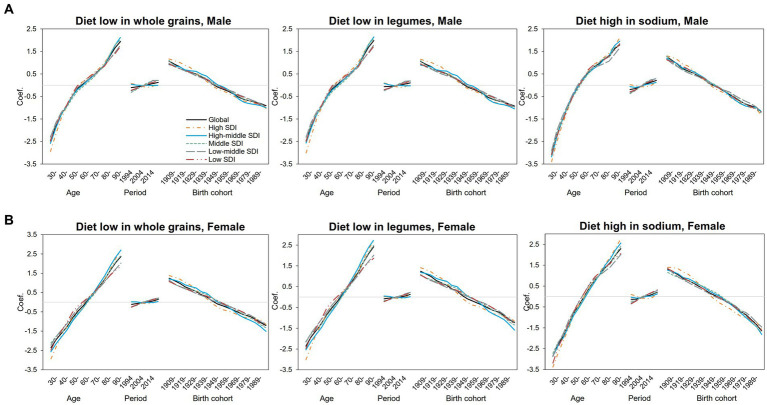
Age–period–cohort-related trends in IHD mortality from 1990 to 2019 globally and SDI quintiles attributable to most common dietary risks stratified by sex **(A)** Male **(B)** Female.

We further separately analyzed the age, period, and cohort effects of the three major dietary factors (diet low in whole grains, diet low in legumes, and diet high in sodium) in different SDI regions (Supplementary Tables S2–S7). The age, period, and cohort effects on DALYs and mortality attributed to three dietary factors were globally similar, with slightly different separation trends for different SDI regions and dietary factors, and relatively larger fluctuations in DALYs can be seen (Figure 2). As shown in Figure 3, DALYs caused by low levels of whole-grains intake increased with age, while the period effect did not change significantly, and the cohort effect showed an overall downward trend.

The risk of DALYs attributable to diet with low whole-grains intake increased significantly with age in both men (Figure 3A) and women (Figure 3B). For male people, the risk of attributable DALYs with age slowed down from 55 to 75 years old and then increased again. In female people, however, age-related DALY risk has been increasing almost linearly compared with that of male people. The risk of DALY in high- and high-middle-SDI areas decreased slightly over the years, but in middle-SDI areas and below, the DALY risk increased slightly since 2009. The period effect trends were not significant in both genders. Specifically, in middle-SDI regions, men in 2019 had the highest DALY risk attributable to low levels of whole-grains intake, with the RR and 95% CI of 1.13 (1.09, 1.18), an increase of 34% compared with 1994 (Supplementary Table S5). For cohort effects, the trend decreased linearly with birth year in almost all SDI regions and reached the lowest point in the 1994–1998 birth cohort. For DALYs caused by low whole-grains intake, the RRs of cohort effects continuously decreased globally from 2.00 (1.90, 2.10) in the 1904–1908 cohort to 0.62 (0.47, 0.82) in the 1989–1993 cohort for male people and from 2.38 (2.19, 2.58) to 0.49 (0.30, 0.80) for female people (Supplementary Table S5). As for the diet low in legumes, the effect was similar to that for the diet low in whole grains (Supplementary Table S6).

## Discussion

In this GBD-based study, we revealed patterns and the latest overall trends in mortality, DALYs, associated socio-economic levels, and dietary risk factors related to the occurrence and progression of IHD worldwide. We found that although ASRs of IHD death and DALYs decreased from 1990 to 2019, the absolute number increased by more than half, indicating that IHD still caused a high social burden. Therefore, not only ASR indicators but also the absolute values are equally important for policymakers and health planners when measuring the effectiveness of IHD prevention and treatment in different regions over time.

From a regional perspective, among all SDI regions, the absolute value of IHD burden decreased only in high-SDI areas, which also had the greatest reduction in ASRs. Compared with high-SDI countries, the burden of IHD remains high in middle- and low-SDI regions (20). More than 80% of deaths occurred in low-income and lower-middle-income countries (LLMICs) (21, 22), where universal health coverage or access to healthcare services is often limited, resulting in huge economic loss (23). Epidemic and burden trends of IHD vary significantly among countries and regions due to the differences in socioeconomic development level, dietary behavior, and living habits (24, 25). That is, due to the aging of the population, the differences in regional economic development, and the risks faced, the low- and middle-SDI regions will bear an increasing burden. The burden caused by IHD has adverse economic consequences at multiple levels for individuals, families, governments, and society as a whole. Furthermore, not only targeted, effective prevention and treatment strategies are needed, but it must also be judged whether the existing prevention strategies are effective. Therefore, we conducted an in-depth analysis of various factors and found that age, gender, time, and dietary risks were potential causes of increased IHD mortality and DALYs.

### Age effects

The adverse outcome of vascular-related diseases was related to age and gender. The global increase in crude death rate and the decrease in ASDR of IHD since 1990 can be attributed to the change of population structure, especially the aging of the social population, and it may also be related to the advancements of global healthcare. According to the World Population Prospects, the proportion of the global population aged 65 and over is expected to rise from 10 to 16% from 2022 to 2050 (26).

Consistent with previous studies, when other variables remained unchanged, the risk of IHD burden among people aged 55 and above was significantly increased compared to the younger population, both globally and in each SDI region (8). According to the APC analysis, the risk of IHD burden increased with age, that is, the oldest age group had the highest risk of death and DALYs. In terms of regional differences, the most significant age effect was concentrated in high-middle-SDI regions, which also indicates a significant gap between developed and developing countries in early death intervention (3).

Although both genders suffer from similar IHD-related risks, the risk of adverse outcomes was higher in men than in women. In contrast to women, the risk of IHD burden continued to increase with age, while the trend slowed down for men in the middle-aged group, especially for DALY risks, with more significant differences. This may be related to the decrease of estrogen level in elderly women after menopause and the disappearance of the protective effect of estrogen on the heart (27). In addition to biological factors, gender differences can also be explained from the perspective of differences in many behavioral risk factors (3, 28). The risk of IHD morbidity and adverse outcomes increased in both genders after the age of 55.

### Period effects

With the rapid development of the social economy, environmental changes, and the improvement of living conditions, it can be assumed that the risk of adverse outcomes of IHD should be reduced (29). In the mixed-effect model, it can be seen that IHD mortality and DALY rates decreased with years, which is consistent with this assumption. However, after controlling for age and birth cohort effects, the overall period effect showed a slight upward trend in the risk of death and DALYs. Considering regional differences in development rates, further differentiation of the subsamples from different SDI regions revealed that the risk of IHD burden decreased in both high- and high-middle-SDI areas. This suggests that in high-income North America, Australia, Europe, and other areas with high social and economic development levels, transient external environmental changes—such as the improvement of screening technology, which facilitates the early detection and treatment of IHD patients and thus the improvement of disease diagnosis and treatment that can reduce the risk of death from IHD—may have beneficial effects on the progression of disease and the occurrence of adverse outcomes. In addition, factors such as the increase in life expectancy *per capita* may also prove beneficial.

Correspondingly, in low-income Africa and other regions with low- or middle-SDI levels, the period effect increased the risk of IHD mortality and DALY, partly reflecting the insufficient screening, diagnosis, and treatment of IHD in these regions (30). Moreover, the expected reduction in period effects may also be influenced by environmental deterioration and strong cohort effects. On the other hand, the overall fluctuation range of the period effect value is relatively small, and there are not too many groups, which may increase the instability of the results. Of course, the overall fluctuation range of the period effect value is small, and the period grouping is small, which may increase the data instability. Therefore, this interpretation can only be used as a relevant reference.

### Cohort effects

The cohort effect was more obvious compared with the period effect, and the overall risk was gradually reduced in different regions. Disease burden was high in birth cohorts before 1949. The earliest birth cohort (1904–1908) had the highest risk coefficient, which also reflected that the quality of the latest birth cohort was relatively high regardless of the regional economic development level. However, it should also be noted that the cohort effects for people in high-SDI regions with higher levels of economic development decreased at a relatively earlier stage than that of people in lower-SDI areas, suggesting a negative correlation between social development and risk outcomes.

Those born in the early years experienced more social unrest, such as the Russo-Japanese War in 1904, World War I in 1914, the October Revolution in Russia in 1917, and the May Fourth Movement in China in 1919. By the early 1950s, capitalist economic development in the West ushered in a golden age. The United Nations was established in 1945, many African countries became independent in 1960, the European Union was formed in 1993, and so on. Such social stability and sustainable development have greatly improved the quality of the birth population and reduced the risk of adverse disease outcomes.

### Diet-related risk factors

The most effective strategy for reducing the burden of IHD is early prevention targeting the risk factors. In the early stages of IHD, prevention measures targeting various risk factors should be carried out at the population level. IHD risk factors include metabolic, behavioral, and environmental factors. Studies have suggested that IHD-related mortality in recent years is largely attributed to metabolic risk factors, such as high systolic blood pressure (SBP) and high low-density lipoprotein (LDL) cholesterol (3, 31). Our previous study also reached this conclusion (8). In addition, we observed 23 level 3 risk factors for IHD, indicating that high SBP, high LDL cholesterol, and a diet low in nuts and seeds are the top three risk factors. The trend of IHD burden attributed to risk factors in recent years suggests that intervention and management of risk factors are effective. Although metabolic risk factors have been emphasized in many studies, considering that dietary behavior is modifiable and universally applicable, dietary risks should not be ignored.

Balanced diet is a well-known consensus, but poor dietary habits still affect disease burden and its changing trends in IHD patients (32). Dietary control is of universal importance in all regions, but prevention policies should be tailored to priorities and local dietary habits. The majority of level 2 risk factors related to IHD DALYs were accounted for by dietary risks among behavioral risk factors, suggesting a need for concerted efforts to address dietary risks in order to effectively reduce the burden of IHD globally. The first step is to understand the differences of dietary attribution burden in different regions. Different from the traditional tertiary prevention, the Blue Book on the prevention and treatment of cardiovascular diseases in China focuses on “balanced diet, reasonable exercise, smoking cessation, healthy psychology, healthy sleep and healthy environment” as its zero-level prevention in view of the pressure of disease prevention and control. Similarly, not only in China, but in many developing regions, the situation of early prevention is not optimistic.

Almost all countries recommend adequate intake of foods such as vegetables, fruits, beans, and legumes in their dietary guidelines. IHD burden attributable to poor dietary habits of low whole grains, low legumes, and low nuts and seeds has shown similar trends over the past 30 years. In high-SDI regions such as high-income North America, this burden declined to the lowest point by 2019. Previous studies demonstrated that whole grains, such as oats and wheat, improved CVD to some extent (30). People with type 2 diabetes will experience health benefits if they consume more than 60 g of whole grains per day (33). In the early stage, most developed countries with high- and high-middle-SDI were based on animal food, which can also be called excessive nutritional diet structure, to provide high energy, high fat, and high protein, but this contained low dietary fiber content, leading to higher risk of CVD. However, since the late 1960s, the CVD mortality in Western developed countries has shown a significant downward trend, which is attributed to the improvement of health concepts and primary prevention measures, that is, the effective control of risk factors. For example, Finland, a country with high SDI, was once the country with the highest CVD incidence in the MONICA Project report (34). Since 1972, Finland has implemented the North Karelia Project with a series of policy-led community participation projects including increasing the availability of low-fat foods, effectively improving the lifestyle and diet structure of its residents and reducing their IHD mortality rate. Lee S. et al. (35) reported that the structure and quality of IHD-related diets among adults in the US had improved during the 20 years since 1980, and the intake of whole grains, fruits, and vegetables increased.

In contrast, in low- and low-middle-SDI regions such as South Asia, the attributable burden caused by insufficient intake of legumes and nuts/seeds has had no significant difference over the years. Studies have shown that CVD has become a major and growing cause of death and disability in Southeast Asia, and CVD has become one of the top ten causes of death in countries such as Bangladesh (36–38). The 2018 Astana Declaration highlighted the important role of primary care in strengthening health systems, and the WHO also provided cost-effective interventions. In middle-SDI countries such as Brazil, increased investment in national primary care since the early 1990s has somewhat reduced the risk of death from IHD (39, 40). However, in many low- and middle-income countries such as Bangladesh, investment in primary healthcare is still lacking (40, 41), accompanied by unhealthy dietary patterns (22), which has not achieved ideal results.

This study has certain limitations. Firstly, the data in our study came from the GBD dataset, and thus all limitations of the GBD methodology were also applicable to this study. In some countries, IHD-related data sources were not available, and their results were derived only from the GBD modeling process. However, these regions also tend to have relatively high disease burden, which may hinder the estimation of actual local IHD burden and may have further affected our findings. Our secondary analysis of regional differences based on SDI indicators can reduce this deficiency to some extent. Secondly, we only focused on the IHD burden attributable to diet-related risk factors and did not examine other risk factors. Since the attributable risks of IHD were assumed to be independent, this may inflate our estimated PAF values. On the other hand, it makes it feasible for us to analyze dietary factors separately. Although dietary factors are not the most important risk factors for IHD, considering that dietary behavior is modifiable and universally applicable, it is meaningful to explore dietary risk factors alone on the basis of such a large number of bases. Thirdly, we did not estimate results for units smaller than the country, nor did we conduct a more detailed analysis of the different subtypes of IHD disease.

## Conclusion

In summary, our study systematically delineated the burden of IHD from 1990 to 2019. Diet-related attributable burden suggested that effective control of these selected risk factors is required for effective IHD management. In addition, the results of different regions show that the national conditions and development realities of different countries should be combined. Therefore, the regional classification used in the GBD study can also be used as the regional classification for IHD prevention. Developing countries with heavy IHD burden can learn from the experience of relatively more developed countries in the same region to address what has been described as “our generation’s social justice issue,” to further promote health equity and social justice, so as to formulate more effective and targeted cardiovascular protection and treatment strategies, effectively implement effective health education and health promotion, and reduce the risk caused by controllable adverse behaviors.

## Data availability statement

Publicly available datasets were analyzed in this study. This data can be found at: http://ghdx.healthdata.org/gbd-results-tool.

## Ethics statement

The list of all data sources used in GBD 2019 is publicly available at the Global Health Data Exchange website (http://ghdx.healthdata.org/gbd-results-tool); therefore, ethical proof is not applicable to this study.

## Author contributions

FW, CY, and YZ: conceptualization and methodology. FW and SM: software and visualization. FW: writing—original draft preparation. FW, SM, YZ, WS, and CY: writing—review and editing. All authors have read and agreed to publish the final manuscript.

## Funding

This research was funded by the National Natural Science Foundation of China (Grant No. 72204211), the Natural Science Fund for Colleges and Universities in Jiangsu Province (No. 22KJD310005), and the Outstanding Talent Research Initiation Foundation of Xuzhou Medical University (No. D2021036). The funders had no role in the study design, data collection and analysis, decision to publish, or preparation of the manuscript.

## Conflict of interest

The authors declare that the research was conducted in the absence of any commercial or financial relationships that could be construed as a potential conflict of interest.

## Publisher’s note

All claims expressed in this article are solely those of the authors and do not necessarily represent those of their affiliated organizations, or those of the publisher, the editors and the reviewers. Any product that may be evaluated in this article, or claim that may be made by its manufacturer, is not guaranteed or endorsed by the publisher.

## Footnotes

^1^ https://vizhub.healthdata.org/gbd-results/
