# Supplementary material for: Risk assessment of dietary factors in global pattern of ischemic heart disease mortality and disability-adjusted life years over 30 years
Source: Front Nutr. 2023 Jun 14;10:1151445. doi: 10.3389/fnut.2023.1151445 (PMC10300343; doi:10.3389/fnut.2023.1151445)
Supplement: Supplementary file 1 [file Data_Sheet_1.PDF]

Figure S1 The age-standardized death rate of global ischemic heart disease for both sexes, 1990–2019.

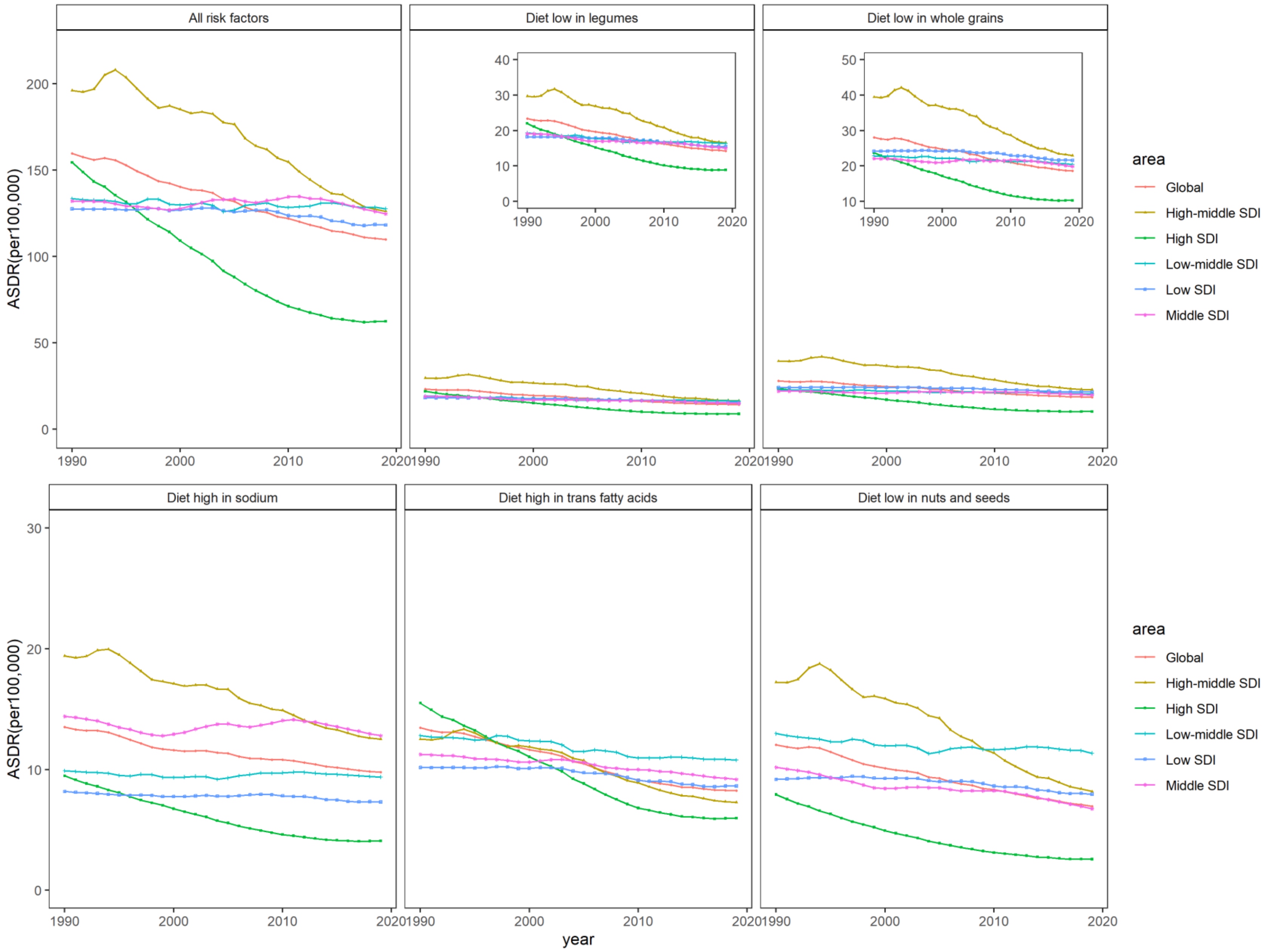

Table S1 Mixed effect model estimates (RR, 95% CIs) predicting the risk of DALYs for ischemic heart disease (IHD), globally and by SDI region separately

Value	Global	High SDI	High-Middle SDI	Middle SDI	Low- Middle SDI	Low SDI
**Age group (year)**						
10-54	1(reference)	1(reference)	1(reference)	1(reference)	1(reference)	1(reference)
≥55	**217.46(74.74,632.53)**	**18.84(5.28,67.26)**	**1801.6(375.49,8644.05)**	**627.66(270.59,1456.07)**	**103.86(44.77,241.02)**	**47.02(21.56,102.55)**
**Sex**						
Female	1(reference)	1(reference)	1(reference)	1(reference)	1(reference)	1(reference)
Male	**13.9(4.78,40.44)**	**5.08(1.42,18.13)**	**65.64(13.68,314.94)**	**17.17(7.4,39.84)**	**6.75(2.91,15.68)**	**3.25(1.49,7.09)**
**Year**						
1990	1(reference)	1(reference)	1(reference)	1(reference)	1(reference)	1(reference)
1999	0.59(0.30,1.16)	**0.34(0.15,0.75)**	0.73(0.27,1.95)	0.68(0.40,1.16)	0.76(0.45,1.29)	0.91(0.56,1.49)
2009	0.33(0.17,0.65)	**0.13(0.06,0.29)**	**0.23(0.09,0.63)**	0.66(0.39,1.13)	0.64(0.37,1.08)	0.76(0.47,1.25)
2019	0.23(0.12,0.45)	**0.10(0.04,0.22)**	**0.09(0.03,0.25)**	**0.51(0.30,0.87)**	**0.58(0.34,0.99)**	**0.57(0.35,0.92)**
**Risks**						
Diet high in sodium	1(reference)	1(reference)	1(reference)	1(reference)	1(reference)	1(reference)
Diet high in TFA	2.00(0.54,7.39)	1.23(0.26,5.86)	3.61(0.53,24.62)	2.29(0.82,6.43)	1.50(0.53,4.20)	1.22(0.47,3.18)
Diet low in legumes	1.61(0.44,5.95)	1.02(0.21,4.84)	2.35(0.34,16.06)	1.95(0.70,5.46)	1.34(0.48,3.76)	0.97(0.37,2.52)
Diet low in nuts and seeds	2.13(0.58,7.89)	1.53(0.32,7.25)	3.32(0.49,22.63)	2.41(0.86,6.74)	1.45(0.52,4.06)	1.27(0.49,3.31)
Diet low in whole grains	1.38(0.37,5.11)	0.92(0.19,4.35)	1.81(0.27,12.38)	1.78(0.64,4.99)	1.12(0.40,3.13)	0.81(0.31,2.11)
**Risk age interaction**						
≥55×Diet high in TFA	0.3(0.07,1.37)	2.99(0.49,18.08)	**0.03(0.01,0.28)**	**0.09(0.03,0.30)**	1.34(0.41,4.41)	1.50(0.50,4.50)
≥55×Diet low in legumes	**6.25(1.38,28.31)**	**16.58(2.74,100.29)**	5.94(0.65,54.54)	1.04(0.32,3.42)	13.99(4.25,45.97)	**41.47(13.77,124.92)**
≥55×Diet low in nuts and seeds	**0.17(0.04,0.77)**	0.32(0.05,1.94)	**0.10(0.01,0.91)**	**0.04(0.01,0.12)**	1.47(0.45,4.83)	0.92(0.31,2.79)
≥55×Diet low in whole grains	**43.55(9.62,197.18)**	**31.14(5.15,188.35)**	**178.00(19.38,1635.20)**	**5.91(1.80,19.42)**	**76.02(23.11,249.87)**	**667.69(221.66,2011.27)**
**Risk sex interaction**						
Male×Diet high in TFA	0.27(0.06,1.23)	0.96(0.16,5.78)	**0.05(0.01,0.48)**	**0.18(0.05,0.59)**	0.65(0.20,2.13)	0.92(0.31,2.77)
Male×Diet low in legumes	0.74(0.16,3.37)	1.96(0.32,11.85)	0.32(0.03,2.91)	0.42(0.13,1.39)	1.33(0.40,4.36)	2.39(0.79,7.20)
Male×Diet low in nuts and seeds	0.23(0.05,1.05)	0.40(0.07,2.42)	**0.08(0.01,0.70)**	**0.15(0.04,0.48)**	0.76(0.23,2.49)	0.90(0.30,2.71)
Male×Diet low in whole grains	1.50(0.33,6.79)	3.02(0.50,18.29)	1.04(0.11,9.57)	0.73(0.22,2.38)	2.61(0.79,8.58)	**5.11(1.70,15.39)**
**AIC**	847.27	869.04	894.92	817.73	817.76	808.28
**BIC**	894.91	916.68	942.56	865.37	865.4	855.92

Note: DALYs: Disability-Adjusted Life Years, SDI: Social-demographic index, TFA: trans fatty acids, AIC: Akaike information criterion, BIC: Bayesian information criterion, CI: confidence intervals, RR: relative risk.

Table S2 The relative risks of ischemic heart disease (IHD) mortality attributable to low whole grain diets due to age, period, and cohort effects, globally and each SDI regions separately

value	Male						Female					
	Global	High SDI	High-middle SDI	Middle SDI	Low-middle SDI	Low SDI	Global	High SDI	High-middle SDI	Middle SDI	Low-middle SDI	Low SDI
**Age**												
25-29	0.09(0.03,0.28)	0.05(0.01,0.24)	0.08(0,1.22)	0.1(0.01,1.32)	0.1(0.02,0.41)	0.08(0.04,0.15)	0.09(0.02,0.55)	0.05(0,0.59)	0.08(0,15.04)	0.12(0.03,0.56)	0.11(0.02,0.57)	0.08(0.05,0.14)
30-34	0.16(0.09,0.29)	0.11(0.06,0.22)	0.15(0.04,0.53)	0.18(0.05,0.66)	0.19(0.1,0.37)	0.17(0.13,0.22)	0.15(0.05,0.4)	0.1(0.03,0.32)	0.12(0.01,2.28)	0.17(0.07,0.43)	0.17(0.07,0.43)	0.15(0.12,0.19)
35-39	0.27(0.19,0.39)	0.23(0.16,0.34)	0.27(0.12,0.58)	0.28(0.12,0.67)	0.3(0.19,0.47)	0.28(0.23,0.33)	0.2(0.1,0.42)	0.18(0.08,0.37)	0.17(0.02,1.29)	0.21(0.1,0.42)	0.24(0.13,0.46)	0.23(0.19,0.27)
40-44	0.39(0.3,0.52)	0.39(0.29,0.52)	0.4(0.22,0.72)	0.38(0.19,0.75)	0.41(0.29,0.58)	0.41(0.36,0.47)	0.28(0.16,0.48)	0.28(0.16,0.49)	0.24(0.05,1.06)	0.26(0.15,0.45)	0.33(0.2,0.54)	0.36(0.32,0.41)
45-49	0.61(0.49,0.76)	0.65(0.52,0.82)	0.61(0.38,0.97)	0.55(0.32,0.94)	0.62(0.47,0.82)	0.68(0.61,0.75)	0.44(0.3,0.66)	0.48(0.31,0.72)	0.37(0.12,1.14)	0.41(0.28,0.6)	0.5(0.34,0.73)	0.59(0.54,0.65)
50-54	0.86(0.72,1.03)	0.95(0.79,1.16)	0.82(0.56,1.22)	0.76(0.49,1.18)	0.9(0.72,1.13)	0.98(0.9,1.07)	0.62(0.45,0.85)	0.7(0.5,0.98)	0.53(0.22,1.27)	0.57(0.42,0.77)	0.71(0.53,0.96)	0.81(0.75,0.88)
55-59	1.04(0.9,1.21)	1.17(1,1.38)	1(0.73,1.38)	0.93(0.65,1.34)	1.09(0.9,1.31)	1.16(1.08,1.25)	0.82(0.64,1.06)	0.91(0.68,1.2)	0.75(0.38,1.48)	0.74(0.58,0.94)	0.94(0.74,1.2)	1.03(0.97,1.1)
60-64	1.28(1.13,1.44)	1.42(1.24,1.63)	1.24(0.96,1.61)	1.16(0.87,1.54)	1.3(1.12,1.51)	1.4(1.32,1.49)	1.16(0.95,1.41)	1.26(1,1.59)	1.12(0.66,1.92)	1.07(0.89,1.29)	1.25(1.04,1.52)	1.3(1.24,1.37)
65-69	1.55(1.4,1.7)	1.71(1.52,1.91)	1.52(1.23,1.89)	1.42(1.13,1.78)	1.55(1.37,1.75)	1.65(1.57,1.73)	1.59(1.36,1.86)	1.68(1.39,2.03)	1.67(1.06,2.62)	1.48(1.28,1.72)	1.59(1.37,1.86)	1.69(1.62,1.76)
70-74	1.93(1.78,2.1)	2.1(1.9,2.32)	1.9(1.56,2.3)	1.86(1.55,2.24)	1.88(1.7,2.08)	2.03(1.95,2.12)	2.27(1.97,2.61)	2.33(1.98,2.75)	2.57(1.64,4.02)	2.16(1.89,2.45)	2.09(1.83,2.38)	2.25(2.17,2.34)
75-79	2.46(2.26,2.66)	2.66(2.41,2.94)	2.46(2.01,3.02)	2.52(2.11,3)	2.32(2.11,2.55)	2.33(2.23,2.42)	3.25(2.79,3.78)	3.37(2.86,3.97)	3.9(2.3,6.62)	3.16(2.74,3.64)	2.85(2.49,3.26)	2.95(2.84,3.07)
80-84	3.6(3.28,3.95)	3.92(3.51,4.38)	3.74(2.95,4.75)	3.9(3.19,4.76)	3.2(2.87,3.56)	3.27(3.12,3.42)	5.12(4.26,6.16)	5.56(4.6,6.7)	6.49(3.35,12.59)	5.29(4.44,6.3)	4.09(3.49,4.81)	4.03(3.84,4.22)
85-89	5.27(4.7,5.9)	5.81(5.1,6.63)	5.93(4.43,7.94)	6.04(4.7,7.76)	4.31(3.78,4.92)	4.1(3.88,4.33)	7.69(6.11,9.68)	9.44(7.51,11.85)	10.22(4.49,23.27)	7.98(6.39,9.97)	5.51(4.5,6.74)	5.22(4.92,5.53)
90-94	6.97(6.06,8.02)	8.28(7.07,9.7)	8.4(5.9,11.97)	7.48(5.47,10.23)	5.94(5.05,7)	5.58(5.21,5.97)	10.59(7.98,14.05)	14.31(10.85,18.87)	14.8(5.46,40.11)	11.17(8.49,14.71)	7.52(5.87,9.65)	6.37(5.94,6.84)
**Period**												
1994	0.9(0.83,0.98)	1.07(0.98,1.18)	1.04(0.86,1.27)	0.73(0.6,0.89)	0.78(0.7,0.86)	0.8(0.77,0.83)	0.89(0.76,1.05)	1.02(0.87,1.19)	1.02(0.62,1.68)	0.78(0.67,0.91)	0.77(0.66,0.89)	0.79(0.76,0.82)
1999	0.93(0.89,0.98)	1.03(0.97,1.08)	1(0.89,1.12)	0.83(0.74,0.94)	0.86(0.8,0.91)	0.87(0.85,0.89)	0.93(0.85,1.03)	1.01(0.92,1.11)	1.01(0.75,1.36)	0.85(0.77,0.93)	0.87(0.8,0.95)	0.87(0.85,0.89)
2004	0.96(0.95,0.98)	0.95(0.93,0.97)	0.98(0.94,1.02)	0.98(0.94,1.02)	0.94(0.91,0.96)	0.97(0.96,0.98)	0.96(0.93,0.99)	0.95(0.92,0.98)	0.97(0.88,1.07)	1(0.97,1.03)	0.98(0.95,1.01)	0.97(0.97,0.98)
2009	1.01(1,1.03)	0.91(0.89,0.92)	1(0.96,1.04)	1.11(1.06,1.16)	1.05(1.02,1.07)	1.07(1.06,1.08)	1.01(0.97,1.04)	0.92(0.89,0.95)	0.98(0.89,1.09)	1.09(1.05,1.12)	1.05(1.02,1.09)	1.05(1.05,1.06)
2014	1.08(1.02,1.13)	0.95(0.9,1.01)	0.99(0.88,1.11)	1.22(1.08,1.37)	1.24(1.17,1.32)	1.13(1.1,1.16)	1.06(0.97,1.17)	0.97(0.88,1.07)	0.98(0.73,1.32)	1.17(1.07,1.28)	1.17(1.07,1.28)	1.14(1.11,1.17)
2019	1.13(1.04,1.23)	1.1(1.01,1.21)	0.99(0.82,1.21)	1.25(1.02,1.52)	1.23(1.11,1.37)	1.22(1.17,1.27)	1.16(0.99,1.36)	1.15(0.98,1.35)	1.04(0.63,1.71)	1.19(1.02,1.39)	1.24(1.07,1.43)	1.24(1.2,1.29)
**Cohort**												
1904-1908	2.92(2.34,3.63)	3.21(2.44,4.22)	2.49(1.44,4.32)	3.02(1.85,4.91)	2.65(2.03,3.46)	2.55(2.29,2.84)	3.45(2.3,5.16)	3.96(2.51,6.26)	3.31(0.79,13.92)	3.01(2.06,4.39)	3.11(2.16,4.48)	2.91(2.6,3.24)
1909-1913	2.55(2.09,3.1)	2.98(2.3,3.86)	2.38(1.43,3.95)	2.33(1.51,3.6)	2.29(1.8,2.91)	2.32(2.1,2.56)	3.05(2.12,4.38)	3.67(2.41,5.6)	3.13(0.83,11.84)	2.51(1.79,3.51)	2.57(1.86,3.57)	2.56(2.31,2.83)
1914-1918	2.27(1.89,2.72)	2.74(2.15,3.5)	2.26(1.4,3.63)	2.08(1.41,3.06)	2.09(1.67,2.6)	2.11(1.93,2.31)	2.64(1.9,3.68)	3.28(2.21,4.87)	2.86(0.82,9.99)	2.27(1.67,3.07)	2.24(1.66,3.01)	2.24(2.04,2.47)
1919-1923	1.95(1.65,2.32)	2.43(1.92,3.08)	1.98(1.25,3.11)	1.87(1.31,2.67)	1.89(1.54,2.32)	1.92(1.76,2.09)	2.2(1.62,2.99)	2.8(1.92,4.08)	2.44(0.74,8.06)	2.06(1.56,2.71)	1.99(1.51,2.62)	2.02(1.84,2.21)
1924-1928	1.79(1.52,2.11)	2.17(1.72,2.74)	1.89(1.21,2.96)	1.72(1.23,2.42)	1.71(1.4,2.09)	1.79(1.64,1.94)	1.96(1.46,2.64)	2.41(1.67,3.48)	2.25(0.69,7.28)	1.92(1.47,2.5)	1.79(1.38,2.32)	1.83(1.67,2)
1929-1933	1.64(1.39,1.94)	1.75(1.38,2.22)	1.86(1.18,2.91)	1.63(1.16,2.28)	1.52(1.25,1.86)	1.59(1.46,1.73)	1.75(1.3,2.36)	1.89(1.3,2.73)	2.08(0.64,6.83)	1.76(1.35,2.3)	1.57(1.21,2.05)	1.63(1.49,1.79)
1934-1938	1.46(1.22,1.73)	1.44(1.13,1.84)	1.63(1.02,2.61)	1.5(1.05,2.15)	1.39(1.13,1.72)	1.44(1.32,1.57)	1.47(1.08,2.01)	1.48(1.01,2.17)	1.72(0.5,5.9)	1.62(1.22,2.15)	1.41(1.07,1.86)	1.49(1.36,1.64)
1939-1943	1.28(1.06,1.55)	1.18(0.92,1.53)	1.49(0.91,2.46)	1.33(0.9,1.97)	1.25(1,1.57)	1.3(1.18,1.43)	1.32(0.94,1.85)	1.2(0.8,1.81)	1.58(0.43,5.83)	1.47(1.08,2)	1.27(0.94,1.72)	1.36(1.23,1.5)
1944-1948	1.06(0.86,1.31)	0.98(0.74,1.29)	1.22(0.71,2.09)	1.13(0.72,1.75)	1.08(0.84,1.39)	1.15(1.04,1.28)	1.07(0.73,1.55)	0.93(0.6,1.45)	1.25(0.31,5.11)	1.25(0.88,1.76)	1.1(0.79,1.53)	1.22(1.09,1.35)
1949-1953	0.92(0.73,1.16)	0.85(0.63,1.14)	1.02(0.57,1.83)	0.98(0.59,1.61)	0.96(0.73,1.27)	1.02(0.91,1.14)	0.91(0.6,1.38)	0.78(0.48,1.26)	1.01(0.22,4.64)	1.08(0.73,1.59)	0.98(0.67,1.42)	1.08(0.96,1.21)
1954-1958	0.84(0.65,1.08)	0.77(0.56,1.06)	0.93(0.49,1.77)	0.87(0.5,1.53)	0.87(0.64,1.19)	0.89(0.78,1)	0.83(0.52,1.32)	0.68(0.4,1.14)	0.9(0.17,4.74)	0.95(0.61,1.48)	0.89(0.58,1.35)	0.94(0.83,1.07)
1959-1963	0.76(0.58,1.01)	0.71(0.5,1)	0.82(0.41,1.66)	0.8(0.42,1.5)	0.81(0.57,1.14)	0.78(0.68,0.9)	0.74(0.44,1.25)	0.63(0.36,1.13)	0.77(0.12,4.75)	0.84(0.51,1.39)	0.81(0.51,1.3)	0.81(0.71,0.93)
1964-1968	0.68(0.49,0.93)	0.62(0.43,0.9)	0.68(0.31,1.47)	0.71(0.35,1.45)	0.75(0.51,1.1)	0.7(0.6,0.82)	0.66(0.37,1.19)	0.57(0.3,1.08)	0.64(0.09,4.79)	0.73(0.42,1.28)	0.74(0.43,1.25)	0.72(0.62,0.84)
1969-1973	0.58(0.41,0.83)	0.54(0.36,0.82)	0.53(0.22,1.28)	0.6(0.27,1.35)	0.65(0.43,1)	0.63(0.53,0.75)	0.58(0.3,1.13)	0.5(0.25,1.02)	0.51(0.05,5.03)	0.61(0.32,1.16)	0.65(0.36,1.19)	0.66(0.56,0.78)
1974-1978	0.53(0.35,0.81)	0.49(0.31,0.79)	0.47(0.16,1.32)	0.54(0.21,1.4)	0.59(0.36,0.98)	0.58(0.48,0.7)	0.52(0.23,1.16)	0.46(0.2,1.04)	0.44(0.03,6.94)	0.52(0.23,1.14)	0.56(0.27,1.15)	0.58(0.48,0.7)
1979-1983	0.5(0.29,0.85)	0.48(0.27,0.85)	0.44(0.12,1.65)	0.5(0.15,1.65)	0.53(0.28,1)	0.5(0.39,0.65)	0.48(0.17,1.37)	0.44(0.15,1.27)	0.4(0.01,14.82)	0.45(0.16,1.28)	0.52(0.21,1.27)	0.48(0.38,0.62)
1984-1988	0.48(0.23,0.99)	0.45(0.19,1.02)	0.42(0.07,2.69)	0.47(0.09,2.33)	0.49(0.21,1.15)	0.46(0.32,0.66)	0.42(0.09,1.88)	0.41(0.09,1.91)	0.35(0,65.85)	0.38(0.09,1.63)	0.44(0.12,1.61)	0.4(0.28,0.58)
1989-1993	0.44(0.12,1.58)	0.43(0.09,1.99)	0.4(0.02,10.82)	0.42(0.03,6.5)	0.44(0.1,1.88)	0.42(0.22,0.78)	0.35(0.03,4.26)	0.4(0.03,5.34)	0.28(0,2485.68)	0.31(0.03,3.18)	0.37(0.04,3.29)	0.33(0.17,0.64)
1994-1998	0.4(0.01,10.83)	0.36(0.003,37.26)	0.37(0,2564.72)	0.38(0,313.01)	0.38(0.01,20.01)	0.39(0.07,2.04)	0.3(0,99.75)	0.32(0,478.72)	0.22(0,1.8*10^9)	0.27(0,46.57)	0.32(0,54.16)	0.28(0.05,1.59)

Table S3 The relative risks of ischemic heart disease (IHD) mortality attributable to low legumes diets due to age, period, and cohort effects, globally and each SDI regions separately

value	Male						Female					
	Global	High SDI	High-middle SDI	Middle SDI	Low-middle SDI	Low SDI	Global	High SDI	High-middle SDI	Middle SDI	Low-middle SDI	Low SDI
**Age**												
25-29	0.08(0.01,0.52)	0.05(0,1.09)	0.08(0,1.43)	0.1(0,2.82)	0.1(0.02,0.49)	0.08(0.04,0.17)	0.09(0.01,0.75)	0.05(0,21.48)	0.08(0,26.64)	0.12(0.01,1.47)	0.12(0.02,0.88)	0.08(0.05,0.14)
30-34	0.16(0.07,0.39)	0.11(0.03,0.4)	0.15(0.04,0.57)	0.18(0.03,0.95)	0.2(0.1,0.41)	0.17(0.12,0.23)	0.15(0.05,0.48)	0.09(0.01,1.69)	0.12(0,3.21)	0.17(0.04,0.75)	0.18(0.06,0.55)	0.15(0.12,0.2)
35-39	0.27(0.16,0.48)	0.22(0.1,0.48)	0.27(0.12,0.61)	0.28(0.09,0.86)	0.3(0.18,0.5)	0.28(0.23,0.35)	0.2(0.09,0.47)	0.17(0.03,1.02)	0.17(0.02,1.62)	0.21(0.07,0.66)	0.25(0.11,0.55)	0.23(0.19,0.27)
40-44	0.39(0.26,0.61)	0.38(0.21,0.67)	0.4(0.21,0.74)	0.38(0.16,0.92)	0.42(0.28,0.62)	0.41(0.35,0.49)	0.28(0.15,0.53)	0.28(0.07,1.03)	0.23(0.04,1.23)	0.27(0.11,0.65)	0.35(0.19,0.63)	0.35(0.31,0.4)
45-49	0.61(0.43,0.85)	0.64(0.4,1.02)	0.6(0.36,0.99)	0.55(0.28,1.1)	0.64(0.47,0.87)	0.68(0.6,0.77)	0.43(0.27,0.69)	0.47(0.17,1.31)	0.36(0.1,1.24)	0.41(0.22,0.78)	0.48(0.3,0.78)	0.59(0.53,0.65)
50-54	0.85(0.64,1.11)	0.94(0.64,1.39)	0.81(0.54,1.22)	0.75(0.43,1.32)	0.91(0.71,1.17)	0.98(0.89,1.09)	0.6(0.41,0.87)	0.69(0.3,1.6)	0.51(0.19,1.33)	0.56(0.34,0.92)	0.68(0.47,0.99)	0.8(0.74,0.87)
55-59	1.03(0.82,1.29)	1.17(0.85,1.62)	0.98(0.7,1.37)	0.92(0.58,1.45)	1.1(0.9,1.35)	1.16(1.06,1.26)	0.8(0.59,1.08)	0.91(0.45,1.82)	0.72(0.34,1.52)	0.73(0.49,1.08)	0.91(0.67,1.22)	1.02(0.96,1.09)
60-64	1.25(1.04,1.5)	1.42(1.09,1.87)	1.22(0.93,1.6)	1.13(0.78,1.63)	1.28(1.08,1.51)	1.38(1.29,1.48)	1.13(0.9,1.43)	1.25(0.71,2.21)	1.09(0.6,1.97)	1.04(0.77,1.41)	1.23(0.98,1.56)	1.3(1.23,1.37)
65-69	1.52(1.31,1.76)	1.71(1.36,2.15)	1.5(1.2,1.88)	1.4(1.04,1.87)	1.49(1.3,1.7)	1.64(1.55,1.73)	1.57(1.3,1.89)	1.69(1.06,2.7)	1.65(1,2.73)	1.45(1.14,1.85)	1.59(1.32,1.92)	1.68(1.6,1.75)
70-74	1.89(1.66,2.15)	2.12(1.73,2.6)	1.88(1.53,2.31)	1.82(1.43,2.32)	1.79(1.6,2.01)	2.01(1.91,2.11)	2.25(1.9,2.66)	2.39(1.58,3.61)	2.58(1.54,4.34)	2.11(1.69,2.63)	2.1(1.78,2.47)	2.24(2.15,2.33)
75-79	2.43(2.14,2.76)	2.74(2.23,3.37)	2.46(1.97,3.06)	2.47(1.95,3.12)	2.24(2.01,2.49)	2.31(2.2,2.42)	3.24(2.69,3.9)	3.51(2.31,5.31)	3.99(2.13,7.46)	3.1(2.42,3.97)	2.82(2.37,3.35)	2.93(2.81,3.06)
80-84	3.66(3.16,4.24)	4.14(3.28,5.23)	3.82(2.94,4.96)	3.89(2.96,5.1)	3.17(2.81,3.58)	3.32(3.14,3.5)	5.27(4.19,6.61)	5.97(3.71,9.62)	6.83(3.1,15.02)	5.3(3.89,7.21)	4.05(3.29,4.99)	4.09(3.89,4.3)
85-89	5.43(4.54,6.49)	6.08(4.6,8.03)	6.08(4.42,8.37)	6.25(4.46,8.77)	4.32(3.73,5.01)	4.14(3.87,4.43)	7.99(6.02,10.62)	9.77(5.49,17.39)	10.82(4.06,28.81)	8.35(5.67,12.31)	5.52(4.26,7.15)	5.29(4.97,5.63)
90-94	7.26(5.83,9.04)	8.8(6.31,12.28)	8.46(5.75,12.44)	7.84(5.15,11.91)	5.96(4.96,7.15)	5.63(5.19,6.1)	11.24(7.94,15.91)	15.19(7.55,30.56)	15.49(4.74,50.64)	11.97(7.43,19.28)	7.46(5.43,10.27)	6.43(5.96,6.94)
**Period**												
1994	0.94(0.82,1.07)	1.1(0.91,1.32)	1.08(0.88,1.33)	0.78(0.6,1.01)	0.81(0.72,0.91)	0.82(0.78,0.86)	0.92(0.76,1.11)	1.02(0.69,1.52)	1.05(0.59,1.86)	0.84(0.65,1.09)	0.8(0.67,0.96)	0.8(0.77,0.84)
1999	0.95(0.88,1.03)	1.04(0.93,1.17)	1.01(0.9,1.15)	0.87(0.74,1.01)	0.87(0.81,0.93)	0.88(0.86,0.91)	0.95(0.85,1.07)	1.02(0.8,1.29)	1.03(0.73,1.45)	0.88(0.75,1.03)	0.89(0.8,0.99)	0.89(0.87,0.91)
2004	0.96(0.93,0.99)	0.95(0.92,0.99)	0.98(0.94,1.02)	0.97(0.92,1.02)	0.95(0.92,0.97)	0.97(0.96,0.98)	0.96(0.93,1)	0.96(0.88,1.04)	0.98(0.87,1.1)	1(0.94,1.05)	0.96(0.92,0.99)	0.98(0.97,0.99)
2009	1(0.97,1.03)	0.9(0.87,0.94)	0.99(0.95,1.03)	1.08(1.02,1.14)	1.04(1.01,1.07)	1.06(1.05,1.08)	0.99(0.96,1.03)	0.92(0.85,1)	0.97(0.87,1.09)	1.06(1.01,1.12)	1.02(0.98,1.06)	1.04(1.04,1.05)
2014	1.05(0.98,1.14)	0.94(0.84,1.05)	0.97(0.86,1.1)	1.18(1.01,1.38)	1.19(1.12,1.28)	1.11(1.08,1.15)	1.05(0.93,1.17)	0.96(0.76,1.22)	0.96(0.68,1.36)	1.12(0.96,1.31)	1.16(1.04,1.29)	1.12(1.1,1.15)
2019	1.11(0.97,1.26)	1.08(0.9,1.3)	0.97(0.79,1.2)	1.19(0.92,1.54)	1.21(1.08,1.35)	1.2(1.14,1.26)	1.14(0.94,1.37)	1.14(0.76,1.69)	1.01(0.57,1.8)	1.14(0.88,1.47)	1.25(1.04,1.5)	1.22(1.17,1.27)
**Cohort**												
1904-1908	2.87(2.04,4.03)	3.16(1.81,5.51)	2.44(1.33,4.46)	2.98(1.57,5.67)	2.59(1.92,3.49)	2.54(2.23,2.89)	3.45(2.11,5.64)	4.09(1.3,12.89)	3.27(0.59,18.06)	2.94(1.53,5.66)	2.9(1.83,4.59)	2.86(2.54,3.22)
1909-1913	2.53(1.85,3.45)	2.94(1.75,4.95)	2.35(1.34,4.12)	2.36(1.33,4.19)	2.26(1.72,2.96)	2.32(2.06,2.61)	3.05(1.96,4.76)	3.74(1.29,10.82)	3.11(0.63,15.32)	2.53(1.4,4.54)	2.48(1.64,3.75)	2.52(2.26,2.8)
1914-1918	2.29(1.72,3.04)	2.76(1.69,4.52)	2.26(1.33,3.83)	2.1(1.25,3.53)	2.05(1.6,2.63)	2.11(1.89,2.36)	2.67(1.78,4.01)	3.35(1.24,9.07)	2.87(0.64,12.93)	2.3(1.35,3.92)	2.2(1.52,3.21)	2.23(2.01,2.47)
1919-1923	1.97(1.51,2.58)	2.46(1.53,3.97)	1.98(1.19,3.29)	1.88(1.16,3.03)	1.83(1.45,2.31)	1.93(1.74,2.13)	2.21(1.51,3.23)	2.83(1.09,7.32)	2.44(0.57,10.38)	2.08(1.27,3.41)	1.98(1.4,2.8)	2.03(1.84,2.24)
1924-1928	1.81(1.39,2.35)	2.24(1.4,3.59)	1.89(1.15,3.12)	1.74(1.1,2.75)	1.65(1.32,2.07)	1.8(1.63,1.99)	1.98(1.37,2.85)	2.46(0.97,6.25)	2.27(0.54,9.44)	1.95(1.21,3.13)	1.78(1.27,2.48)	1.86(1.69,2.05)
1929-1933	1.66(1.27,2.16)	1.8(1.12,2.9)	1.88(1.13,3.12)	1.64(1.04,2.6)	1.53(1.22,1.92)	1.6(1.44,1.77)	1.76(1.22,2.55)	1.92(0.75,4.93)	2.13(0.5,9.02)	1.79(1.11,2.89)	1.59(1.14,2.22)	1.66(1.51,1.83)
1934-1938	1.47(1.11,1.93)	1.5(0.92,2.45)	1.64(0.97,2.78)	1.51(0.93,2.45)	1.43(1.12,1.81)	1.45(1.3,1.61)	1.48(1,2.17)	1.53(0.58,4.05)	1.75(0.39,7.79)	1.64(0.99,2.72)	1.44(1.01,2.05)	1.51(1.37,1.67)
1939-1943	1.29(0.96,1.74)	1.21(0.72,2.04)	1.53(0.88,2.67)	1.34(0.79,2.27)	1.27(0.98,1.65)	1.3(1.16,1.45)	1.33(0.88,2.01)	1.21(0.43,3.39)	1.65(0.34,7.99)	1.5(0.87,2.6)	1.31(0.9,1.93)	1.37(1.24,1.53)
1944-1948	1.07(0.77,1.48)	1.01(0.58,1.75)	1.23(0.68,2.24)	1.13(0.62,2.04)	1.11(0.84,1.47)	1.15(1.02,1.31)	1.07(0.68,1.7)	0.93(0.31,2.84)	1.29(0.24,6.99)	1.25(0.68,2.3)	1.14(0.75,1.74)	1.23(1.09,1.37)
1949-1953	0.92(0.64,1.32)	0.86(0.48,1.57)	1.03(0.54,1.97)	0.97(0.5,1.88)	0.98(0.72,1.34)	1.02(0.89,1.16)	0.91(0.55,1.52)	0.78(0.23,2.6)	1.04(0.17,6.44)	1.06(0.54,2.09)	1.02(0.63,1.64)	1.08(0.95,1.22)
1954-1958	0.84(0.57,1.26)	0.79(0.41,1.51)	0.96(0.47,1.95)	0.86(0.41,1.82)	0.88(0.62,1.25)	0.89(0.76,1.03)	0.82(0.47,1.45)	0.68(0.18,2.54)	0.93(0.13,6.79)	0.92(0.43,1.97)	0.92(0.54,1.56)	0.94(0.82,1.08)
1959-1963	0.77(0.5,1.2)	0.72(0.36,1.46)	0.85(0.39,1.83)	0.79(0.34,1.83)	0.82(0.56,1.2)	0.78(0.66,0.92)	0.74(0.39,1.4)	0.64(0.15,2.71)	0.8(0.09,6.95)	0.83(0.35,1.95)	0.82(0.46,1.49)	0.81(0.7,0.94)
1964-1968	0.68(0.42,1.11)	0.63(0.29,1.35)	0.69(0.3,1.61)	0.71(0.28,1.82)	0.75(0.49,1.15)	0.7(0.58,0.84)	0.66(0.33,1.36)	0.57(0.12,2.79)	0.65(0.06,7.05)	0.73(0.28,1.91)	0.76(0.39,1.48)	0.72(0.61,0.85)
1969-1973	0.58(0.33,1.01)	0.54(0.23,1.24)	0.53(0.2,1.38)	0.6(0.21,1.75)	0.65(0.41,1.05)	0.64(0.52,0.78)	0.58(0.26,1.32)	0.5(0.09,2.91)	0.5(0.03,7.58)	0.61(0.2,1.83)	0.67(0.32,1.43)	0.66(0.55,0.79)
1974-1978	0.53(0.28,1.02)	0.47(0.18,1.23)	0.46(0.15,1.44)	0.54(0.15,1.91)	0.6(0.34,1.04)	0.58(0.46,0.74)	0.52(0.2,1.39)	0.44(0.06,3.47)	0.44(0.02,11.38)	0.52(0.14,1.99)	0.57(0.23,1.41)	0.58(0.47,0.71)
1979-1983	0.5(0.22,1.14)	0.45(0.14,1.46)	0.44(0.1,1.84)	0.5(0.1,2.42)	0.53(0.26,1.08)	0.5(0.37,0.68)	0.48(0.14,1.7)	0.43(0.03,5.85)	0.39(0.01,27.81)	0.46(0.08,2.63)	0.51(0.16,1.61)	0.48(0.37,0.63)
1984-1988	0.47(0.15,1.49)	0.42(0.08,2.34)	0.42(0.06,3.12)	0.46(0.05,3.93)	0.49(0.19,1.27)	0.46(0.3,0.7)	0.41(0.07,2.58)	0.39(0.01,18.28)	0.34(0,158.83)	0.38(0.03,4.59)	0.43(0.08,2.29)	0.4(0.27,0.59)
1989-1993	0.43(0.06,3.21)	0.4(0.02,10.41)	0.39(0.01,14.36)	0.41(0.01,16.94)	0.43(0.09,2.22)	0.41(0.19,0.88)	0.34(0.02,7.47)	0.38(0,279.92)	0.27(0,13614.83)	0.3(0,18.65)	0.35(0.02,5.92)	0.33(0.16,0.66)
1994-1998	0.4(0,72.86)	0.36(0,4277.86)	0.35(0,6931)	0.38(0,3502.57)	0.38(0,34.94)	0.39(0.05,2.87)	0.29(0,374.05)	0.31(0,3.5*10^7)	0.2(0,2.1*10^10)	0.26(0,2695.52)	0.3(0,207.82)	0.27(0.04,1.78)

Table S4 The relative risks of ischemic heart disease (IHD) mortality attributable to high sodium diets due to age, period, and cohort effects, globally and each SDI regions separately

value	Male						Female					
	Global	High SDI	High-middle SDI	Middle SDI	Low-middle SDI	Low SDI	Global	High SDI	High-middle SDI	Middle SDI	Low-middle SDI	Low SDI
**Age**												
25-29	0.05(0,1.16)	0.03(0,183.89)	0.04(0,10.34)	0.05(0,297.25)	0.06(0,1.38)	0.05(0.01,0.38)	0.06(0,2.12)	0.03(0,98.92)	0.06(0,38.23)	0.07(0,4.44)	0.06(0,4.06)	0.04(0,0.57)
30-34	0.11(0.03,0.38)	0.08(0,2.2)	0.1(0.01,0.82)	0.11(0,3.47)	0.13(0.04,0.44)	0.1(0.04,0.24)	0.1(0.02,0.58)	0.07(0,2.45)	0.1(0,2.56)	0.11(0.01,0.89)	0.11(0.02,0.74)	0.09(0.03,0.25)
35-39	0.21(0.1,0.45)	0.17(0.02,1.18)	0.2(0.06,0.71)	0.21(0.03,1.78)	0.24(0.11,0.52)	0.19(0.12,0.32)	0.15(0.05,0.49)	0.12(0.01,1.1)	0.14(0.02,1.29)	0.16(0.04,0.66)	0.17(0.05,0.6)	0.15(0.08,0.29)
40-44	0.37(0.21,0.66)	0.32(0.07,1.37)	0.38(0.15,0.99)	0.35(0.07,1.79)	0.4(0.22,0.72)	0.34(0.23,0.5)	0.26(0.12,0.59)	0.22(0.05,1.05)	0.24(0.05,1.1)	0.25(0.09,0.69)	0.32(0.13,0.77)	0.28(0.17,0.45)
45-49	0.62(0.38,1)	0.6(0.18,2)	0.63(0.28,1.4)	0.58(0.15,2.22)	0.66(0.41,1.08)	0.59(0.43,0.81)	0.45(0.24,0.85)	0.41(0.12,1.43)	0.41(0.13,1.32)	0.44(0.2,0.96)	0.49(0.24,1.02)	0.48(0.32,0.71)
50-54	0.93(0.62,1.39)	0.97(0.35,2.71)	0.94(0.48,1.84)	0.84(0.27,2.6)	1.01(0.67,1.53)	0.94(0.72,1.23)	0.66(0.4,1.11)	0.68(0.24,1.91)	0.59(0.23,1.51)	0.62(0.33,1.16)	0.78(0.43,1.42)	0.8(0.57,1.11)
55-59	1.3(0.93,1.83)	1.35(0.57,3.23)	1.29(0.73,2.28)	1.22(0.47,3.13)	1.39(0.98,1.96)	1.28(1.02,1.6)	1(0.66,1.51)	1.02(0.44,2.39)	0.89(0.42,1.88)	0.94(0.57,1.56)	1.17(0.72,1.91)	1.17(0.89,1.55)
60-64	1.76(1.33,2.33)	1.87(0.9,3.88)	1.69(1.05,2.71)	1.67(0.77,3.65)	1.83(1.37,2.44)	2.01(1.66,2.42)	1.53(1.09,2.13)	1.59(0.79,3.21)	1.36(0.74,2.48)	1.45(0.97,2.16)	1.78(1.2,2.65)	1.84(1.47,2.32)
65-69	2.25(1.78,2.84)	2.5(1.35,4.63)	2.08(1.4,3.09)	2.26(1.19,4.27)	2.24(1.77,2.84)	2.44(2.09,2.85)	2.17(1.63,2.88)	2.28(1.26,4.14)	2(1.18,3.38)	2.14(1.53,3)	2.37(1.71,3.28)	2.53(2.09,3.07)
70-74	2.58(2.11,3.15)	2.86(1.66,4.94)	2.42(1.72,3.41)	2.67(1.56,4.57)	2.37(1.94,2.89)	2.81(2.46,3.21)	2.86(2.18,3.75)	3.21(1.85,5.56)	2.91(1.69,5.01)	2.81(2.02,3.91)	2.77(2.07,3.72)	3.19(2.69,3.78)
75-79	3.16(2.62,3.82)	3.42(2,5.84)	3.14(2.26,4.36)	3.37(2.04,5.54)	2.62(2.18,3.14)	3.08(2.72,3.49)	3.99(2.94,5.41)	4.68(2.62,8.37)	4.49(2.35,8.59)	3.87(2.63,5.68)	3.4(2.51,4.6)	4.07(3.43,4.84)
80-84	3.54(2.89,4.35)	4.16(2.32,7.47)	3.78(2.64,5.39)	3.48(2.03,5.96)	2.93(2.41,3.56)	3.74(3.27,4.28)	5.07(3.5,7.34)	6.61(3.36,13.01)	6.06(2.7,13.57)	4.83(3,7.77)	4.12(2.89,5.89)	4.91(4.04,5.97)
85-89	5.01(3.94,6.38)	5.78(2.91,11.46)	5.76(3.78,8.76)	5.33(2.8,10.13)	3.9(3.1,4.9)	4.67(3.99,5.47)	7.32(4.65,11.52)	10.17(4.49,23.02)	9.32(3.45,25.14)	7.4(4.1,13.37)	5.51(3.57,8.52)	6.51(5.16,8.21)
90-94	5.99(4.48,8.01)	8.1(3.58,18.32)	7.04(4.25,11.66)	6.11(2.79,13.38)	5.24(3.97,6.92)	6.26(5.17,7.57)	9.9(5.72,17.13)	15.91(5.96,42.41)	12.9(3.91,42.54)	11.07(5.41,22.65)	7.47(4.4,12.67)	7.79(5.89,10.31)
**Period**												
1994	0.83(0.69,0.99)	1.03(0.64,1.63)	0.94(0.69,1.27)	0.69(0.42,1.14)	0.71(0.59,0.85)	0.75(0.66,0.84)	0.88(0.66,1.16)	1.1(0.66,1.85)	0.97(0.55,1.72)	0.8(0.56,1.14)	0.73(0.55,0.99)	0.7(0.6,0.83)
1999	0.86(0.77,0.96)	0.98(0.74,1.3)	0.91(0.76,1.09)	0.79(0.58,1.06)	0.8(0.71,0.89)	0.82(0.76,0.88)	0.88(0.74,1.04)	1.02(0.74,1.39)	0.93(0.66,1.31)	0.82(0.66,1.02)	0.83(0.7,0.99)	0.81(0.74,0.89)
2004	0.94(0.91,0.98)	0.94(0.85,1.03)	0.95(0.89,1.01)	0.95(0.86,1.06)	0.94(0.9,0.97)	0.94(0.92,0.97)	0.94(0.89,0.99)	0.93(0.83,1.03)	0.95(0.85,1.06)	0.99(0.92,1.06)	0.95(0.9,1.01)	0.95(0.92,0.98)
2009	1.06(1.02,1.09)	0.93(0.85,1.02)	1.03(0.97,1.09)	1.14(1.03,1.27)	1.11(1.07,1.15)	1.1(1.07,1.12)	1.02(0.96,1.08)	0.89(0.8,0.99)	1.01(0.9,1.13)	1.1(1.02,1.18)	1.07(1.01,1.14)	1.09(1.06,1.13)
2014	1.14(1.03,1.27)	0.99(0.75,1.3)	1.07(0.89,1.28)	1.28(0.95,1.73)	1.27(1.14,1.41)	1.19(1.11,1.28)	1.1(0.93,1.3)	0.95(0.69,1.29)	1.03(0.73,1.45)	1.17(0.94,1.46)	1.21(1.01,1.45)	1.22(1.11,1.34)
2019	1.24(1.03,1.48)	1.15(0.72,1.84)	1.13(0.84,1.53)	1.32(0.8,2.18)	1.34(1.12,1.61)	1.32(1.18,1.49)	1.23(0.93,1.63)	1.14(0.68,1.92)	1.13(0.64,2)	1.2(0.83,1.72)	1.33(0.99,1.78)	1.38(1.18,1.62)
**Cohort**												
1904-1908	3.56(2.06,6.14)	3.67(0.72,18.59)	3.29(1.28,8.44)	3.62(0.85,15.44)	3.15(1.84,5.41)	3.08(2.2,4.32)	3.63(1.48,8.9)	4.05(0.68,24.13)	3.52(0.5,24.59)	3.16(1.01,9.87)	3.87(1.59,9.38)	3.81(2.28,6.35)
1909-1913	3.02(1.81,5.03)	3.48(0.74,16.42)	2.91(1.2,7.08)	2.81(0.73,10.81)	2.62(1.58,4.35)	2.83(2.07,3.86)	3.25(1.4,7.59)	4.01(0.73,22)	3.28(0.51,20.87)	2.78(0.95,8.11)	3.07(1.34,7.02)	3.33(2.05,5.41)
1914-1918	2.57(1.58,4.18)	3.11(0.69,13.99)	2.61(1.12,6.1)	2.37(0.67,8.42)	2.29(1.42,3.7)	2.6(1.94,3.49)	2.84(1.26,6.37)	3.77(0.73,19.48)	2.96(0.5,17.63)	2.5(0.9,6.97)	2.57(1.17,5.61)	2.87(1.8,4.57)
1919-1923	2.07(1.3,3.32)	2.57(0.59,11.24)	2.15(0.94,4.89)	2.02(0.6,6.83)	1.97(1.24,3.12)	2.32(1.75,3.07)	2.37(1.08,5.2)	3.23(0.65,16.13)	2.48(0.43,14.19)	2.26(0.84,6.11)	2.21(1.04,4.69)	2.46(1.57,3.87)
1924-1928	1.89(1.19,3.01)	2.35(0.54,10.23)	2.04(0.9,4.61)	1.82(0.55,6.02)	1.74(1.1,2.74)	2.06(1.56,2.71)	2.14(0.98,4.66)	2.86(0.58,14.18)	2.34(0.41,13.28)	2.1(0.79,5.62)	1.94(0.92,4.06)	2.12(1.35,3.32)
1929-1933	1.71(1.07,2.74)	1.94(0.44,8.59)	1.85(0.81,4.22)	1.7(0.51,5.71)	1.59(1,2.52)	1.75(1.33,2.31)	1.89(0.86,4.14)	2.28(0.45,11.52)	2.05(0.35,11.88)	1.93(0.71,5.21)	1.69(0.8,3.58)	1.83(1.16,2.88)
1934-1938	1.52(0.94,2.47)	1.6(0.35,7.38)	1.62(0.69,3.8)	1.56(0.44,5.48)	1.46(0.9,2.35)	1.56(1.17,2.08)	1.63(0.72,3.66)	1.83(0.35,9.64)	1.77(0.29,10.77)	1.76(0.63,4.92)	1.5(0.69,3.26)	1.6(1,2.56)
1939-1943	1.32(0.79,2.2)	1.3(0.26,6.4)	1.41(0.58,3.46)	1.38(0.36,5.23)	1.29(0.78,2.13)	1.36(1,1.85)	1.41(0.6,3.3)	1.42(0.25,8.04)	1.54(0.23,10.09)	1.6(0.55,4.69)	1.32(0.58,2.98)	1.41(0.87,2.31)
1944-1948	1.1(0.64,1.9)	1.06(0.2,5.66)	1.19(0.46,3.08)	1.17(0.28,4.9)	1.12(0.65,1.91)	1.18(0.84,1.64)	1.19(0.48,2.93)	1.09(0.18,6.72)	1.3(0.18,9.4)	1.39(0.44,4.34)	1.15(0.48,2.74)	1.23(0.73,2.06)
1949-1953	0.97(0.54,1.74)	0.88(0.15,5.19)	1.01(0.36,2.81)	1.05(0.22,4.94)	0.97(0.54,1.74)	1.01(0.71,1.45)	1.05(0.4,2.76)	0.86(0.13,5.95)	1.13(0.14,9.25)	1.23(0.36,4.17)	1(0.39,2.56)	1.07(0.61,1.85)
1954-1958	0.87(0.46,1.64)	0.78(0.12,5.12)	0.91(0.3,2.72)	0.91(0.17,4.91)	0.87(0.46,1.63)	0.88(0.6,1.31)	0.94(0.34,2.65)	0.73(0.09,5.67)	1.01(0.11,9.52)	1.06(0.28,3.93)	0.89(0.32,2.47)	0.94(0.52,1.7)
1959-1963	0.76(0.38,1.51)	0.71(0.1,5.27)	0.77(0.23,2.51)	0.78(0.12,4.88)	0.8(0.4,1.58)	0.77(0.5,1.19)	0.8(0.26,2.44)	0.63(0.07,5.69)	0.83(0.08,9.16)	0.87(0.21,3.59)	0.81(0.27,2.43)	0.83(0.44,1.56)
1964-1968	0.66(0.31,1.39)	0.6(0.07,5.12)	0.63(0.17,2.26)	0.68(0.09,5.05)	0.74(0.35,1.54)	0.67(0.42,1.07)	0.7(0.21,2.33)	0.55(0.05,5.78)	0.69(0.05,9.08)	0.75(0.16,3.45)	0.73(0.22,2.42)	0.71(0.36,1.4)
1969-1973	0.57(0.26,1.29)	0.51(0.05,5.07)	0.52(0.13,2.1)	0.6(0.07,5.31)	0.63(0.28,1.41)	0.56(0.34,0.93)	0.61(0.16,2.23)	0.46(0.04,5.79)	0.57(0.04,9.3)	0.62(0.12,3.29)	0.64(0.17,2.35)	0.59(0.28,1.22)
1974-1978	0.49(0.2,1.22)	0.46(0.04,5.73)	0.44(0.09,2.1)	0.5(0.04,5.75)	0.55(0.23,1.35)	0.48(0.27,0.85)	0.48(0.11,2.11)	0.39(0.02,6.75)	0.45(0.02,10.52)	0.49(0.07,3.19)	0.51(0.12,2.22)	0.47(0.21,1.06)
1979-1983	0.43(0.15,1.29)	0.4(0.02,8.3)	0.4(0.06,2.58)	0.43(0.02,8.01)	0.49(0.17,1.42)	0.42(0.21,0.84)	0.42(0.07,2.59)	0.36(0.01,12.64)	0.36(0.01,20.26)	0.4(0.04,4.23)	0.46(0.08,2.63)	0.4(0.15,1.04)
1984-1988	0.4(0.09,1.85)	0.37(0,28.39)	0.38(0.03,5.45)	0.38(0.01,22.47)	0.45(0.11,1.85)	0.39(0.15,1.03)	0.33(0.02,5.78)	0.32(0,72.65)	0.29(0,170.57)	0.3(0.01,12.53)	0.38(0.03,5.33)	0.32(0.07,1.36)
1989-1993	0.38(0.02,6.27)	0.36(0,1172.88)	0.38(0,50.98)	0.35(0,617.62)	0.39(0.03,5.58)	0.34(0.05,2.2)	0.26(0,47.09)	0.3(0,3789.91)	0.24(0,18355.66)	0.23(0,185.31)	0.29(0,45.81)	0.26(0.02,3.92)
1994-1998	0.31(0,3496.08)	0.27(0,1.3*10^12)	0.32(0,4.0*10^6)	0.29(0,8.9*10^9)	0.31(0,3033.34)	0.31(0,82.08)	0.19(0,7.6*10^6)	0.21(0,8.4*10^12)	0.16(0,6.5*10^13)	0.18(0,3.4*10^7)	0.23(0,5.2*10^5)	0.2(0,1446.03)

Table S5 The relative risks of DALYs for ischemic heart disease (IHD) attributable to low whole grain diets due to age, period, and cohort effects, globally and each SDI regions separately

value	Male, RR (95%CI)						Female, RR (95%CI)					
	Global	High SDI	High-middle SDI	Middle SDI	Low-middle SDI	Low SDI	Global	High SDI	High-middle SDI	Middle SDI	Low-middle SDI	Low SDI
**Age**												
25-29	0.15(0.12,0.2)	0.1(0.06,0.16)	0.14(0.07,0.27)	0.19(0.12,0.28)	0.18(0.13,0.25)	0.15(0.12,0.18)	0.17(0.12,0.23)	0.1(0.05,0.19)	0.14(0.05,0.41)	0.22(0.15,0.31)	0.2(0.13,0.29)	0.15(0.13,0.18)
30-34	0.28(0.25,0.32)	0.2(0.15,0.25)	0.26(0.19,0.36)	0.31(0.24,0.38)	0.33(0.27,0.39)	0.29(0.26,0.31)	0.25(0.21,0.31)	0.17(0.12,0.25)	0.2(0.11,0.39)	0.3(0.24,0.37)	0.29(0.23,0.37)	0.26(0.24,0.28)
35-39	0.45(0.41,0.49)	0.38(0.33,0.44)	0.44(0.36,0.55)	0.46(0.39,0.54)	0.49(0.43,0.55)	0.46(0.43,0.49)	0.33(0.29,0.39)	0.29(0.23,0.37)	0.28(0.17,0.45)	0.35(0.29,0.42)	0.4(0.33,0.48)	0.38(0.35,0.4)
40-44	0.61(0.57,0.65)	0.6(0.54,0.67)	0.62(0.52,0.73)	0.58(0.51,0.67)	0.63(0.57,0.7)	0.64(0.61,0.67)	0.44(0.39,0.49)	0.44(0.37,0.52)	0.37(0.26,0.53)	0.41(0.36,0.48)	0.52(0.45,0.6)	0.56(0.54,0.59)
45-49	0.88(0.84,0.93)	0.94(0.86,1.03)	0.88(0.77,1)	0.8(0.72,0.89)	0.9(0.83,0.97)	0.98(0.95,1.02)	0.64(0.59,0.7)	0.69(0.6,0.79)	0.55(0.42,0.72)	0.6(0.54,0.67)	0.73(0.66,0.82)	0.86(0.83,0.89)
50-54	1.16(1.11,1.21)	1.28(1.19,1.38)	1.11(1,1.24)	1.03(0.95,1.12)	1.22(1.14,1.3)	1.32(1.28,1.36)	0.84(0.78,0.91)	0.94(0.84,1.05)	0.73(0.59,0.9)	0.77(0.71,0.84)	0.97(0.88,1.05)	1.1(1.07,1.13)
55-59	1.29(1.24,1.33)	1.45(1.36,1.54)	1.23(1.13,1.35)	1.15(1.07,1.24)	1.34(1.26,1.41)	1.43(1.4,1.47)	1.02(0.96,1.08)	1.13(1.03,1.23)	0.93(0.79,1.1)	0.92(0.86,0.99)	1.16(1.08,1.25)	1.27(1.24,1.3)
60-64	1.42(1.37,1.46)	1.58(1.5,1.66)	1.38(1.28,1.48)	1.28(1.21,1.36)	1.43(1.37,1.5)	1.55(1.51,1.58)	1.29(1.23,1.35)	1.41(1.31,1.51)	1.25(1.1,1.43)	1.18(1.12,1.25)	1.38(1.3,1.47)	1.44(1.41,1.47)
65-69	1.51(1.47,1.55)	1.67(1.6,1.74)	1.48(1.39,1.57)	1.38(1.31,1.45)	1.5(1.44,1.56)	1.6(1.57,1.63)	1.55(1.49,1.61)	1.65(1.56,1.75)	1.63(1.47,1.81)	1.44(1.38,1.51)	1.54(1.47,1.62)	1.64(1.61,1.66)
70-74	1.61(1.57,1.65)	1.76(1.69,1.82)	1.58(1.5,1.67)	1.55(1.49,1.61)	1.56(1.51,1.61)	1.69(1.66,1.72)	1.89(1.83,1.95)	1.96(1.86,2.06)	2.14(1.94,2.37)	1.78(1.72,1.85)	1.72(1.65,1.8)	1.87(1.84,1.89)
75-79	1.69(1.66,1.73)	1.84(1.77,1.91)	1.7(1.61,1.8)	1.72(1.66,1.79)	1.6(1.55,1.65)	1.6(1.58,1.63)	2.23(2.16,2.3)	2.32(2.21,2.44)	2.68(2.38,3.01)	2.15(2.07,2.24)	1.94(1.86,2.02)	2.02(1.99,2.05)
80-84	1.98(1.94,2.03)	2.16(2.07,2.25)	2.07(1.94,2.21)	2.13(2.05,2.21)	1.77(1.71,1.83)	1.81(1.78,1.84)	2.81(2.7,2.92)	3.03(2.87,3.21)	3.54(3.06,4.09)	2.87(2.75,3.01)	2.24(2.14,2.34)	2.21(2.17,2.25)
85-89	2.3(2.24,2.36)	2.53(2.41,2.66)	2.59(2.39,2.8)	2.62(2.5,2.74)	1.89(1.82,1.97)	1.8(1.77,1.83)	3.34(3.18,3.5)	4.06(3.79,4.35)	4.39(3.66,5.27)	3.45(3.26,3.66)	2.4(2.27,2.54)	2.28(2.23,2.33)
90-94	2.46(2.38,2.54)	2.9(2.73,3.08)	2.94(2.67,3.24)	2.64(2.5,2.8)	2.1(2.01,2.2)	1.97(1.92,2.01)	3.7(3.49,3.92)	4.93(4.53,5.37)	5.09(4.08,6.36)	3.92(3.66,4.21)	2.67(2.49,2.85)	2.24(2.19,2.3)
**Period**												
1994	1(0.98,1.02)	1.17(1.13,1.21)	1.14(1.08,1.2)	0.84(0.81,0.87)	0.87(0.85,0.9)	0.89(0.87,0.9)	0.98(0.95,1.02)	1.11(1.06,1.17)	1.11(0.99,1.24)	0.88(0.85,0.92)	0.88(0.84,0.92)	0.88(0.86,0.89)
1999	0.99(0.97,1)	1.09(1.06,1.11)	1.06(1.02,1.1)	0.89(0.86,0.91)	0.92(0.9,0.94)	0.93(0.92,0.94)	0.99(0.97,1.01)	1.06(1.03,1.1)	1.06(0.99,1.14)	0.91(0.88,0.93)	0.93(0.91,0.96)	0.93(0.92,0.94)
2004	0.99(0.98,1)	0.97(0.96,0.98)	1.03(1.01,1.05)	0.99(0.97,1)	0.95(0.93,0.97)	0.99(0.98,1)	0.99(0.98,1)	0.97(0.96,0.98)	1.01(0.98,1.03)	1.02(1,1.03)	0.98(0.96,1)	0.99(0.99,1)
2009	0.99(0.98,1)	0.9(0.89,0.91)	0.98(0.96,1)	1.06(1.05,1.08)	1.03(1.01,1.05)	1.05(1.04,1.05)	0.99(0.98,1)	0.9(0.89,0.92)	0.97(0.95,1)	1.05(1.04,1.07)	1.02(1,1.04)	1.03(1.02,1.04)
2014	1.01(0.99,1.02)	0.9(0.88,0.92)	0.92(0.89,0.95)	1.13(1.1,1.16)	1.14(1.11,1.16)	1.06(1.05,1.07)	1(0.98,1.02)	0.92(0.89,0.95)	0.92(0.86,0.99)	1.09(1.06,1.12)	1.09(1.06,1.12)	1.07(1.06,1.08)
2019	1.03(1,1.05)	1(0.96,1.03)	0.9(0.85,0.95)	1.13(1.09,1.18)	1.12(1.09,1.16)	1.1(1.09,1.12)	1.05(1.01,1.08)	1.05(1,1.1)	0.94(0.84,1.05)	1.07(1.03,1.12)	1.12(1.07,1.17)	1.12(1.11,1.14)
**Cohort**												
1904-1908	2.0(1.9,2.1)	2.24(2.03,2.47)	1.74(1.51,2)	1.99(1.82,2.18)	1.8(1.67,1.94)	1.75(1.69,1.82)	2.38(2.19,2.58)	2.75(2.4,3.14)	2.32(1.71,3.14)	2.02(1.83,2.22)	2.06(1.86,2.27)	1.98(1.91,2.06)
1909-1913	1.82(1.75,1.91)	2.15(1.96,2.35)	1.72(1.51,1.95)	1.64(1.52,1.77)	1.63(1.52,1.74)	1.65(1.6,1.71)	2.19(2.03,2.35)	2.63(2.33,2.98)	2.26(1.71,2.99)	1.77(1.63,1.93)	1.81(1.66,1.97)	1.82(1.76,1.89)
1914-1918	1.7(1.63,1.77)	2.04(1.87,2.22)	1.68(1.49,1.89)	1.56(1.46,1.67)	1.55(1.47,1.65)	1.58(1.53,1.62)	1.97(1.84,2.1)	2.43(2.18,2.72)	2.13(1.65,2.76)	1.69(1.57,1.82)	1.67(1.54,1.8)	1.67(1.62,1.72)
1919-1923	1.52(1.46,1.57)	1.88(1.73,2.04)	1.52(1.35,1.7)	1.49(1.4,1.58)	1.47(1.39,1.55)	1.5(1.46,1.54)	1.69(1.59,1.79)	2.16(1.94,2.4)	1.86(1.46,2.37)	1.61(1.5,1.71)	1.56(1.46,1.68)	1.57(1.52,1.62)
1924-1928	1.45(1.4,1.5)	1.74(1.61,1.89)	1.51(1.36,1.69)	1.43(1.35,1.52)	1.41(1.34,1.48)	1.44(1.41,1.48)	1.57(1.49,1.66)	1.93(1.74,2.13)	1.79(1.41,2.27)	1.56(1.47,1.66)	1.47(1.38,1.57)	1.48(1.44,1.52)
1929-1933	1.39(1.34,1.44)	1.47(1.36,1.6)	1.57(1.4,1.75)	1.39(1.31,1.47)	1.3(1.23,1.37)	1.34(1.31,1.38)	1.48(1.4,1.56)	1.58(1.43,1.75)	1.74(1.37,2.22)	1.5(1.41,1.59)	1.34(1.26,1.44)	1.38(1.34,1.42)
1934-1938	1.27(1.22,1.32)	1.26(1.16,1.37)	1.42(1.26,1.6)	1.32(1.25,1.41)	1.23(1.17,1.3)	1.27(1.23,1.3)	1.29(1.22,1.38)	1.29(1.16,1.44)	1.5(1.16,1.93)	1.44(1.34,1.53)	1.26(1.17,1.35)	1.32(1.28,1.36)
1939-1943	1.17(1.12,1.22)	1.07(0.98,1.17)	1.37(1.21,1.55)	1.22(1.14,1.31)	1.14(1.07,1.21)	1.19(1.16,1.23)	1.2(1.12,1.28)	1.08(0.96,1.21)	1.43(1.09,1.87)	1.34(1.25,1.45)	1.17(1.08,1.26)	1.25(1.21,1.29)
1944-1948	1.02(0.97,1.07)	0.94(0.85,1.03)	1.17(1.02,1.34)	1.08(1,1.17)	1.04(0.98,1.12)	1.11(1.07,1.14)	1.02(0.95,1.1)	0.89(0.78,1.01)	1.19(0.89,1.6)	1.19(1.09,1.3)	1.07(0.98,1.17)	1.17(1.13,1.21)
1949-1953	0.92(0.87,0.97)	0.85(0.76,0.94)	1.02(0.87,1.18)	0.98(0.9,1.08)	0.96(0.89,1.04)	1.01(0.98,1.05)	0.92(0.84,1)	0.78(0.68,0.9)	1(0.72,1.39)	1.08(0.98,1.19)	0.99(0.89,1.09)	1.08(1.04,1.12)
1954-1958	0.88(0.83,0.93)	0.81(0.72,0.91)	0.98(0.83,1.16)	0.92(0.83,1.02)	0.91(0.83,0.99)	0.92(0.89,0.96)	0.87(0.78,0.95)	0.71(0.61,0.83)	0.94(0.65,1.35)	1(0.89,1.12)	0.93(0.83,1.04)	0.98(0.94,1.02)
1959-1963	0.83(0.78,0.89)	0.77(0.68,0.87)	0.89(0.74,1.07)	0.87(0.77,0.97)	0.88(0.8,0.97)	0.85(0.81,0.89)	0.8(0.72,0.9)	0.69(0.58,0.83)	0.83(0.55,1.24)	0.91(0.8,1.04)	0.88(0.77,1)	0.88(0.84,0.92)
1964-1968	0.77(0.71,0.83)	0.7(0.61,0.81)	0.77(0.62,0.95)	0.81(0.71,0.92)	0.85(0.77,0.94)	0.79(0.76,0.84)	0.75(0.66,0.85)	0.65(0.54,0.79)	0.73(0.46,1.14)	0.83(0.71,0.96)	0.84(0.72,0.97)	0.82(0.78,0.86)
1969-1973	0.69(0.63,0.75)	0.64(0.55,0.75)	0.63(0.5,0.8)	0.71(0.62,0.83)	0.77(0.69,0.87)	0.75(0.71,0.79)	0.69(0.6,0.8)	0.6(0.48,0.75)	0.62(0.36,1.04)	0.72(0.61,0.86)	0.77(0.65,0.91)	0.78(0.73,0.83)
1974-1978	0.66(0.59,0.73)	0.61(0.51,0.73)	0.58(0.43,0.77)	0.67(0.56,0.8)	0.74(0.64,0.85)	0.72(0.67,0.76)	0.64(0.54,0.77)	0.57(0.43,0.74)	0.55(0.29,1.05)	0.64(0.52,0.79)	0.7(0.57,0.85)	0.72(0.67,0.77)
1979-1983	0.64(0.57,0.73)	0.62(0.49,0.77)	0.57(0.39,0.82)	0.64(0.52,0.8)	0.69(0.58,0.82)	0.65(0.6,0.71)	0.62(0.49,0.77)	0.57(0.41,0.8)	0.51(0.22,1.19)	0.58(0.44,0.77)	0.66(0.52,0.85)	0.62(0.57,0.68)
1984-1988	0.64(0.54,0.76)	0.6(0.44,0.83)	0.57(0.34,0.94)	0.62(0.47,0.83)	0.65(0.52,0.82)	0.62(0.55,0.7)	0.56(0.41,0.77)	0.56(0.34,0.9)	0.46(0.14,1.52)	0.52(0.36,0.74)	0.59(0.42,0.84)	0.54(0.47,0.62)
1989-1993	0.62(0.47,0.82)	0.6(0.34,1.05)	0.57(0.24,1.32)	0.58(0.37,0.93)	0.61(0.42,0.88)	0.58(0.48,0.71)	0.49(0.3,0.8)	0.56(0.26,1.2)	0.4(0.06,2.78)	0.43(0.25,0.76)	0.51(0.29,0.89)	0.47(0.37,0.58)
1994-1998	0.58(0.30,1.15)	0.52(0.11,2.55)	0.54(0.06,4.55)	0.54(0.19,1.58)	0.55(0.21,1.41)	0.56(0.35,0.92)	0.44(0.15,1.29)	0.46(0.06,3.49)	0.33(0,33.07)	0.39(0.12,1.26)	0.46(0.13,1.58)	0.41(0.23,0.71)

Table S6 The relative risks of DALYs for ischemic heart disease (IHD) attributable to low legumes diets due to age, period, and cohort effects, globally and each SDI regions separately

value	Male, RR (95%CI)						Female, RR (95%CI)					
	Global	High SDI	High-middle SDI	Middle SDI	Low-middle SDI	Low SDI	Global	High SDI	High-middle SDI	Middle SDI	Low-middle SDI	Low SDI
**Age**												
25-29	0.15(0.11,0.21)	0.09(0.04,0.18)	0.14(0.07,0.27)	0.18(0.11,0.31)	0.18(0.13,0.26)	0.15(0.12,0.18)	0.17(0.12,0.24)	0.09(0.03,0.28)	0.14(0.05,0.42)	0.21(0.13,0.35)	0.21(0.13,0.32)	0.15(0.13,0.18)
30-34	0.28(0.24,0.33)	0.19(0.14,0.26)	0.26(0.19,0.37)	0.31(0.23,0.41)	0.34(0.28,0.41)	0.29(0.26,0.32)	0.25(0.2,0.32)	0.16(0.09,0.29)	0.21(0.11,0.4)	0.3(0.22,0.4)	0.31(0.23,0.4)	0.26(0.24,0.29)
35-39	0.45(0.4,0.5)	0.36(0.3,0.44)	0.45(0.36,0.55)	0.47(0.38,0.57)	0.5(0.44,0.57)	0.46(0.43,0.49)	0.33(0.28,0.4)	0.28(0.19,0.41)	0.28(0.17,0.45)	0.35(0.27,0.45)	0.4(0.33,0.5)	0.38(0.35,0.4)
40-44	0.61(0.56,0.67)	0.58(0.5,0.68)	0.62(0.52,0.73)	0.59(0.5,0.7)	0.65(0.58,0.72)	0.64(0.61,0.68)	0.44(0.38,0.5)	0.43(0.32,0.57)	0.36(0.25,0.53)	0.42(0.34,0.52)	0.54(0.46,0.63)	0.55(0.53,0.58)
45-49	0.88(0.82,0.94)	0.93(0.82,1.04)	0.87(0.76,1)	0.81(0.7,0.92)	0.93(0.86,1.01)	0.99(0.95,1.03)	0.63(0.57,0.7)	0.68(0.54,0.84)	0.53(0.4,0.69)	0.6(0.52,0.7)	0.71(0.63,0.8)	0.85(0.82,0.89)
50-54	1.14(1.08,1.21)	1.26(1.15,1.39)	1.09(0.98,1.22)	1.02(0.91,1.14)	1.23(1.15,1.31)	1.32(1.28,1.36)	0.81(0.75,0.88)	0.93(0.78,1.11)	0.69(0.55,0.86)	0.76(0.67,0.86)	0.92(0.83,1.02)	1.09(1.05,1.12)
55-59	1.27(1.21,1.33)	1.44(1.33,1.57)	1.2(1.1,1.32)	1.14(1.03,1.25)	1.36(1.28,1.44)	1.42(1.39,1.46)	0.99(0.93,1.06)	1.12(0.97,1.3)	0.89(0.75,1.06)	0.9(0.82,1)	1.12(1.03,1.21)	1.26(1.23,1.29)
60-64	1.39(1.33,1.44)	1.58(1.48,1.69)	1.35(1.26,1.46)	1.25(1.16,1.35)	1.41(1.34,1.48)	1.52(1.49,1.56)	1.26(1.19,1.33)	1.4(1.24,1.57)	1.21(1.07,1.38)	1.16(1.07,1.25)	1.36(1.27,1.46)	1.44(1.41,1.47)
65-69	1.48(1.43,1.53)	1.68(1.58,1.77)	1.46(1.37,1.55)	1.36(1.28,1.45)	1.45(1.39,1.51)	1.59(1.56,1.62)	1.52(1.46,1.59)	1.66(1.51,1.83)	1.61(1.44,1.79)	1.41(1.32,1.5)	1.54(1.46,1.63)	1.63(1.6,1.66)
70-74	1.58(1.53,1.62)	1.78(1.69,1.87)	1.57(1.48,1.66)	1.51(1.43,1.6)	1.49(1.44,1.54)	1.67(1.64,1.7)	1.87(1.8,1.94)	2(1.84,2.17)	2.15(1.93,2.39)	1.75(1.66,1.85)	1.73(1.65,1.82)	1.85(1.83,1.88)
75-79	1.67(1.63,1.72)	1.89(1.79,1.99)	1.7(1.6,1.8)	1.69(1.6,1.77)	1.54(1.49,1.59)	1.59(1.56,1.62)	2.22(2.14,2.31)	2.4(2.21,2.61)	2.73(2.41,3.1)	2.11(2,2.24)	1.92(1.83,2.01)	2.01(1.97,2.04)
80-84	2.01(1.95,2.07)	2.28(2.15,2.42)	2.11(1.97,2.26)	2.12(2.01,2.23)	1.75(1.69,1.81)	1.83(1.8,1.86)	2.88(2.75,3.01)	3.25(2.95,3.58)	3.72(3.17,4.36)	2.88(2.69,3.08)	2.21(2.1,2.33)	2.25(2.2,2.29)
85-89	2.37(2.29,2.46)	2.65(2.47,2.84)	2.65(2.44,2.89)	2.71(2.55,2.89)	1.89(1.82,1.97)	1.82(1.78,1.86)	3.47(3.28,3.67)	4.21(3.75,4.74)	4.65(3.81,5.68)	3.61(3.32,3.93)	2.41(2.26,2.57)	2.31(2.26,2.36)
90-94	2.56(2.45,2.68)	3.09(2.84,3.36)	2.96(2.68,3.28)	2.78(2.57,3)	2.11(2.01,2.21)	1.99(1.94,2.04)	3.94(3.67,4.22)	5.27(4.57,6.08)	5.35(4.2,6.82)	4.21(3.79,4.67)	2.64(2.44,2.86)	2.27(2.2,2.33)
**Period**												
1994	1.05(1.02,1.07)	1.21(1.15,1.27)	1.18(1.12,1.25)	0.91(0.86,0.96)	0.92(0.89,0.95)	0.91(0.9,0.93)	1.02(0.98,1.06)	1.13(1.04,1.23)	1.15(1.01,1.29)	0.96(0.9,1.02)	0.92(0.88,0.97)	0.9(0.89,0.91)
1999	1(0.99,1.02)	1.1(1.07,1.13)	1.07(1.03,1.11)	0.92(0.89,0.95)	0.93(0.91,0.95)	0.95(0.94,0.96)	1.01(0.98,1.03)	1.07(1.02,1.13)	1.08(1,1.16)	0.94(0.9,0.97)	0.95(0.92,0.98)	0.95(0.94,0.96)
2004	0.98(0.97,0.99)	0.97(0.96,0.98)	1.02(1,1.04)	0.97(0.95,0.99)	0.95(0.93,0.97)	0.99(0.98,1)	0.99(0.98,1)	0.97(0.95,0.99)	1.01(0.98,1.04)	1.01(0.99,1.03)	0.95(0.93,0.97)	0.99(0.99,1)
2009	0.98(0.97,0.99)	0.89(0.88,0.91)	0.97(0.95,0.99)	1.04(1.01,1.06)	1.02(1,1.04)	1.04(1.03,1.05)	0.98(0.97,0.99)	0.9(0.89,0.92)	0.96(0.94,0.99)	1.03(1.01,1.05)	0.99(0.97,1.01)	1.02(1.01,1.03)
2014	0.99(0.97,1.01)	0.89(0.86,0.91)	0.91(0.87,0.94)	1.09(1.06,1.13)	1.1(1.08,1.13)	1.04(1.03,1.06)	0.98(0.96,1.01)	0.91(0.86,0.96)	0.91(0.84,0.98)	1.05(1.01,1.09)	1.08(1.05,1.12)	1.05(1.04,1.06)
2019	1(0.98,1.03)	0.98(0.94,1.03)	0.88(0.83,0.93)	1.09(1.03,1.14)	1.1(1.07,1.14)	1.08(1.06,1.1)	1.02(0.99,1.07)	1.04(0.95,1.12)	0.92(0.81,1.03)	1.03(0.97,1.09)	1.13(1.08,1.18)	1.1(1.09,1.12)
**Cohort**												
1904-1908	1.95(1.83,2.09)	2.18(1.91,2.49)	1.69(1.46,1.97)	1.95(1.73,2.19)	1.75(1.61,1.89)	1.74(1.67,1.81)	2.35(2.14,2.59)	2.8(2.24,3.5)	2.27(1.63,3.17)	1.96(1.7,2.25)	1.91(1.71,2.14)	1.95(1.87,2.03)
1909-1913	1.8(1.7,1.91)	2.11(1.87,2.38)	1.7(1.48,1.95)	1.65(1.49,1.83)	1.6(1.49,1.72)	1.65(1.59,1.71)	2.18(2.01,2.37)	2.67(2.18,3.27)	2.24(1.65,3.05)	1.78(1.58,2.02)	1.74(1.58,1.92)	1.79(1.72,1.86)
1914-1918	1.72(1.63,1.81)	2.06(1.84,2.31)	1.69(1.48,1.92)	1.59(1.45,1.74)	1.53(1.44,1.63)	1.58(1.53,1.63)	1.99(1.85,2.15)	2.5(2.07,3.01)	2.14(1.61,2.85)	1.73(1.55,1.93)	1.65(1.51,1.8)	1.66(1.6,1.72)
1919-1923	1.54(1.46,1.62)	1.91(1.72,2.14)	1.52(1.35,1.72)	1.5(1.38,1.63)	1.44(1.36,1.52)	1.51(1.46,1.55)	1.71(1.6,1.83)	2.2(1.84,2.62)	1.87(1.42,2.45)	1.63(1.48,1.81)	1.56(1.44,1.69)	1.58(1.53,1.63)
1924-1928	1.47(1.4,1.54)	1.81(1.62,2.01)	1.51(1.34,1.7)	1.45(1.34,1.56)	1.37(1.29,1.45)	1.46(1.42,1.5)	1.59(1.49,1.7)	1.98(1.67,2.36)	1.8(1.38,2.35)	1.59(1.45,1.75)	1.46(1.35,1.58)	1.51(1.46,1.56)
1929-1933	1.4(1.34,1.47)	1.52(1.36,1.69)	1.59(1.41,1.79)	1.4(1.3,1.52)	1.3(1.23,1.38)	1.35(1.32,1.39)	1.49(1.39,1.59)	1.61(1.36,1.92)	1.78(1.36,2.34)	1.53(1.39,1.68)	1.35(1.25,1.46)	1.41(1.36,1.46)
1934-1938	1.28(1.22,1.35)	1.3(1.16,1.46)	1.43(1.26,1.62)	1.33(1.22,1.45)	1.26(1.19,1.34)	1.27(1.24,1.31)	1.3(1.21,1.4)	1.33(1.11,1.59)	1.53(1.15,2.02)	1.46(1.32,1.62)	1.29(1.19,1.4)	1.34(1.29,1.38)
1939-1943	1.18(1.12,1.25)	1.1(0.98,1.24)	1.4(1.23,1.61)	1.23(1.12,1.35)	1.16(1.08,1.23)	1.19(1.15,1.23)	1.21(1.12,1.31)	1.09(0.89,1.32)	1.49(1.1,2.01)	1.37(1.22,1.54)	1.2(1.1,1.32)	1.26(1.21,1.3)
1944-1948	1.03(0.96,1.09)	0.96(0.85,1.1)	1.18(1.02,1.37)	1.08(0.97,1.2)	1.07(1,1.15)	1.11(1.07,1.15)	1.03(0.94,1.13)	0.9(0.72,1.11)	1.22(0.88,1.7)	1.2(1.05,1.36)	1.11(1,1.23)	1.18(1.13,1.22)
1949-1953	0.92(0.86,0.99)	0.87(0.75,1)	1.03(0.87,1.21)	0.97(0.86,1.1)	0.98(0.91,1.07)	1.01(0.97,1.05)	0.92(0.83,1.01)	0.78(0.62,0.99)	1.03(0.72,1.48)	1.06(0.91,1.23)	1.02(0.91,1.15)	1.08(1.04,1.13)
1954-1958	0.88(0.82,0.96)	0.83(0.71,0.97)	1.01(0.84,1.2)	0.9(0.78,1.04)	0.92(0.84,1.01)	0.92(0.88,0.96)	0.86(0.77,0.97)	0.71(0.55,0.93)	0.98(0.66,1.45)	0.96(0.81,1.14)	0.96(0.84,1.09)	0.98(0.93,1.03)
1959-1963	0.84(0.77,0.91)	0.79(0.66,0.93)	0.92(0.75,1.11)	0.86(0.74,1.01)	0.89(0.8,0.98)	0.84(0.8,0.89)	0.8(0.71,0.92)	0.7(0.52,0.93)	0.86(0.56,1.33)	0.9(0.74,1.09)	0.9(0.77,1.04)	0.88(0.83,0.92)
1964-1968	0.77(0.7,0.85)	0.72(0.59,0.86)	0.78(0.63,0.97)	0.81(0.68,0.96)	0.85(0.76,0.95)	0.8(0.75,0.84)	0.76(0.65,0.87)	0.65(0.47,0.9)	0.74(0.45,1.2)	0.82(0.66,1.03)	0.86(0.73,1.02)	0.82(0.77,0.87)
1969-1973	0.69(0.61,0.77)	0.64(0.52,0.79)	0.63(0.49,0.81)	0.71(0.58,0.87)	0.77(0.68,0.88)	0.75(0.71,0.8)	0.69(0.59,0.82)	0.59(0.41,0.85)	0.61(0.34,1.07)	0.72(0.56,0.93)	0.79(0.66,0.96)	0.78(0.73,0.84)
1974-1978	0.66(0.58,0.75)	0.59(0.46,0.75)	0.58(0.42,0.78)	0.67(0.53,0.85)	0.74(0.64,0.86)	0.72(0.67,0.77)	0.65(0.53,0.79)	0.55(0.36,0.85)	0.55(0.27,1.1)	0.65(0.47,0.88)	0.71(0.56,0.89)	0.72(0.67,0.78)
1979-1983	0.64(0.54,0.76)	0.58(0.42,0.78)	0.56(0.38,0.83)	0.64(0.48,0.87)	0.69(0.57,0.83)	0.65(0.59,0.71)	0.62(0.48,0.8)	0.55(0.32,0.97)	0.5(0.2,1.25)	0.59(0.4,0.88)	0.66(0.49,0.88)	0.62(0.56,0.68)
1984-1988	0.63(0.5,0.79)	0.56(0.36,0.88)	0.56(0.33,0.95)	0.62(0.42,0.92)	0.65(0.51,0.83)	0.62(0.54,0.7)	0.55(0.38,0.79)	0.53(0.24,1.18)	0.45(0.13,1.62)	0.51(0.3,0.89)	0.57(0.38,0.87)	0.53(0.46,0.62)
1989-1993	0.61(0.42,0.88)	0.56(0.26,1.24)	0.55(0.22,1.35)	0.57(0.3,1.08)	0.6(0.41,0.89)	0.58(0.47,0.72)	0.47(0.26,0.85)	0.54(0.15,1.96)	0.38(0.04,3.16)	0.42(0.18,1)	0.49(0.25,0.94)	0.46(0.36,0.59)
1994-1998	0.58(0.23,1.45)	0.52(0.06,4.38)	0.51(0.05,5.17)	0.54(0.12,2.39)	0.55(0.2,1.53)	0.56(0.33,0.96)	0.43(0.12,1.52)	0.45(0.02,13.69)	0.3(0,51.32)	0.38(0.06,2.36)	0.44(0.11,1.82)	0.4(0.22,0.74)

Table S7 The relative risks of DALYs for ischemic heart disease (IHD) attributable to high sodium diets due to age, period, and cohort effects, globally and each SDI regions separately

value	Male, RR (95%CI)						Female, RR (95%CI)					
	Global	High SDI	High-middle SDI	Middle SDI	Low-middle SDI	Low SDI	Global	High SDI	High-middle SDI	Middle SDI	Low-middle SDI	Low SDI
**Age**												
25-29	0.08(0.05,0.15)	0.06(0.01,0.4)	0.07(0.02,0.27)	0.09(0.02,0.35)	0.1(0.05,0.21)	0.08(0.04,0.15)	0.1(0.05,0.23)	0.06(0.01,0.45)	0.1(0.02,0.44)	0.12(0.05,0.3)	0.1(0.04,0.28)	0.07(0.03,0.17)
30-34	0.19(0.15,0.24)	0.14(0.06,0.3)	0.17(0.1,0.29)	0.2(0.11,0.35)	0.23(0.17,0.31)	0.17(0.13,0.23)	0.18(0.12,0.27)	0.12(0.04,0.3)	0.16(0.07,0.36)	0.19(0.12,0.32)	0.18(0.11,0.3)	0.15(0.11,0.22)
35-39	0.35(0.3,0.4)	0.28(0.17,0.44)	0.34(0.24,0.46)	0.35(0.25,0.51)	0.4(0.33,0.48)	0.32(0.27,0.38)	0.25(0.19,0.34)	0.2(0.11,0.37)	0.23(0.13,0.41)	0.26(0.18,0.37)	0.28(0.2,0.39)	0.24(0.19,0.31)
40-44	0.57(0.51,0.64)	0.49(0.34,0.7)	0.59(0.47,0.75)	0.55(0.42,0.72)	0.62(0.53,0.72)	0.53(0.46,0.6)	0.41(0.34,0.51)	0.34(0.22,0.52)	0.38(0.26,0.56)	0.39(0.3,0.51)	0.49(0.39,0.62)	0.43(0.36,0.51)
45-49	0.9(0.82,0.99)	0.86(0.65,1.15)	0.92(0.75,1.12)	0.85(0.68,1.06)	0.96(0.85,1.09)	0.86(0.77,0.95)	0.66(0.57,0.77)	0.61(0.43,0.85)	0.6(0.45,0.81)	0.65(0.54,0.79)	0.72(0.6,0.87)	0.7(0.61,0.8)
50-54	1.25(1.15,1.36)	1.3(1.03,1.66)	1.26(1.07,1.49)	1.14(0.94,1.37)	1.36(1.23,1.51)	1.26(1.16,1.38)	0.91(0.8,1.03)	0.93(0.71,1.22)	0.81(0.64,1.03)	0.85(0.73,1)	1.06(0.91,1.24)	1.08(0.97,1.21)
55-59	1.61(1.5,1.72)	1.68(1.37,2.05)	1.59(1.38,1.83)	1.5(1.29,1.76)	1.7(1.56,1.85)	1.57(1.46,1.69)	1.25(1.13,1.38)	1.28(1.02,1.6)	1.12(0.93,1.35)	1.18(1.05,1.34)	1.45(1.28,1.64)	1.45(1.32,1.59)
60-64	1.95(1.85,2.07)	2.08(1.76,2.46)	1.88(1.67,2.11)	1.86(1.63,2.11)	2.02(1.88,2.16)	2.22(2.1,2.35)	1.7(1.57,1.85)	1.77(1.47,2.12)	1.53(1.32,1.77)	1.62(1.47,1.79)	1.97(1.78,2.18)	2.04(1.9,2.2)
65-69	2.2(2.1,2.3)	2.44(2.12,2.81)	2.04(1.85,2.24)	2.2(1.98,2.44)	2.17(2.05,2.3)	2.37(2.26,2.49)	2.12(1.98,2.27)	2.23(1.92,2.6)	1.97(1.73,2.23)	2.09(1.93,2.27)	2.29(2.11,2.49)	2.46(2.31,2.62)
70-74	2.16(2.07,2.24)	2.4(2.12,2.72)	2.03(1.87,2.21)	2.22(2.03,2.42)	1.97(1.87,2.07)	2.34(2.25,2.44)	2.39(2.24,2.55)	2.68(2.33,3.09)	2.43(2.13,2.76)	2.33(2.15,2.52)	2.29(2.13,2.47)	2.64(2.5,2.79)
75-79	2.18(2.1,2.26)	2.37(2.09,2.68)	2.17(2,2.35)	2.3(2.11,2.5)	1.8(1.72,1.89)	2.13(2.05,2.21)	2.74(2.55,2.94)	3.2(2.75,3.73)	3.07(2.63,3.58)	2.63(2.4,2.89)	2.32(2.15,2.5)	2.79(2.64,2.96)
80-84	1.94(1.87,2.03)	2.3(2,2.64)	2.07(1.89,2.27)	1.89(1.72,2.07)	1.62(1.54,1.7)	2.07(1.98,2.16)	2.77(2.53,3.02)	3.59(3,4.29)	3.28(2.7,3.97)	2.61(2.32,2.93)	2.25(2.06,2.47)	2.7(2.53,2.88)
85-89	2.18(2.08,2.29)	2.51(2.13,2.96)	2.5(2.25,2.78)	2.3(2.06,2.57)	1.71(1.61,1.81)	2.05(1.95,2.16)	3.15(2.83,3.52)	4.35(3.5,5.41)	3.96(3.13,5.03)	3.18(2.76,3.67)	2.4(2.15,2.69)	2.83(2.62,3.07)
90-94	2.11(1.98,2.24)	2.81(2.31,3.43)	2.45(2.15,2.79)	2.16(1.89,2.47)	1.86(1.73,1.99)	2.2(2.07,2.35)	3.42(3,3.91)	5.44(4.18,7.08)	4.38(3.29,5.84)	3.85(3.24,4.58)	2.64(2.3,3.02)	2.73(2.48,3.01)
**Period**												
1994	0.92(0.88,0.95)	1.11(0.99,1.24)	1.02(0.95,1.1)	0.8(0.74,0.87)	0.8(0.77,0.84)	0.83(0.8,0.86)	0.96(0.89,1.03)	1.2(1.04,1.38)	1.04(0.91,1.2)	0.9(0.83,0.99)	0.84(0.78,0.91)	0.79(0.74,0.83)
1999	0.91(0.89,0.93)	1.05(0.98,1.12)	0.96(0.92,1.01)	0.84(0.79,0.88)	0.86(0.83,0.88)	0.87(0.85,0.89)	0.93(0.89,0.97)	1.08(0.99,1.17)	0.98(0.9,1.06)	0.88(0.83,0.93)	0.89(0.85,0.93)	0.86(0.83,0.89)
2004	0.97(0.96,0.98)	0.97(0.94,0.99)	0.99(0.97,1.01)	0.96(0.94,0.99)	0.94(0.92,0.96)	0.96(0.95,0.97)	0.97(0.96,0.99)	0.95(0.92,0.98)	0.99(0.96,1.02)	1.01(0.99,1.03)	0.96(0.94,0.98)	0.97(0.96,0.98)
2009	1.03(1.02,1.04)	0.92(0.9,0.95)	1.01(0.99,1.03)	1.09(1.06,1.12)	1.09(1.07,1.11)	1.08(1.07,1.09)	1.01(0.99,1.02)	0.88(0.85,0.91)	1(0.97,1.03)	1.06(1.04,1.08)	1.04(1.01,1.06)	1.07(1.06,1.09)
2014	1.07(1.05,1.1)	0.93(0.87,1)	1(0.95,1.05)	1.19(1.12,1.25)	1.17(1.13,1.21)	1.12(1.09,1.14)	1.04(0.99,1.08)	0.89(0.82,0.97)	0.97(0.89,1.06)	1.09(1.03,1.15)	1.13(1.08,1.18)	1.14(1.11,1.18)
2019	1.12(1.08,1.16)	1.04(0.93,1.16)	1.02(0.95,1.1)	1.2(1.1,1.31)	1.22(1.16,1.27)	1.19(1.15,1.24)	1.11(1.03,1.18)	1.04(0.9,1.2)	1.03(0.89,1.18)	1.09(0.99,1.19)	1.2(1.11,1.29)	1.24(1.17,1.31)
**Cohort**												
1904-1908	2.43(2.19,2.71)	2.59(1.8,3.73)	2.3(1.83,2.88)	2.34(1.85,2.97)	2.12(1.86,2.42)	2.13(1.91,2.36)	2.52(2.05,3.09)	2.82(1.8,4.41)	2.49(1.6,3.88)	2.13(1.64,2.76)	2.56(2.06,3.18)	2.6(2.2,3.07)
1909-1913	2.16(1.96,2.38)	2.52(1.78,3.56)	2.1(1.7,2.6)	1.95(1.57,2.42)	1.85(1.64,2.09)	2.02(1.84,2.23)	2.34(1.93,2.83)	2.88(1.88,4.39)	2.38(1.57,3.61)	1.97(1.54,2.52)	2.16(1.77,2.63)	2.38(2.04,2.78)
1914-1918	1.92(1.75,2.11)	2.31(1.65,3.21)	1.95(1.6,2.38)	1.78(1.45,2.17)	1.71(1.53,1.91)	1.94(1.78,2.12)	2.11(1.76,2.53)	2.79(1.86,4.18)	2.2(1.48,3.27)	1.87(1.48,2.36)	1.91(1.59,2.3)	2.13(1.84,2.47)
1919-1923	1.61(1.47,1.76)	1.97(1.42,2.72)	1.65(1.36,2)	1.61(1.33,1.94)	1.54(1.38,1.71)	1.81(1.66,1.96)	1.82(1.53,2.16)	2.47(1.67,3.66)	1.89(1.28,2.78)	1.77(1.42,2.21)	1.73(1.45,2.07)	1.91(1.65,2.2)
1924-1928	1.54(1.41,1.68)	1.88(1.36,2.59)	1.64(1.36,1.98)	1.52(1.26,1.83)	1.44(1.3,1.6)	1.67(1.54,1.81)	1.71(1.44,2.03)	2.28(1.55,3.36)	1.86(1.27,2.72)	1.71(1.38,2.13)	1.59(1.34,1.89)	1.72(1.49,1.97)
1929-1933	1.45(1.33,1.58)	1.63(1.18,2.26)	1.55(1.28,1.88)	1.46(1.21,1.77)	1.36(1.22,1.51)	1.49(1.37,1.61)	1.58(1.32,1.87)	1.91(1.29,2.83)	1.7(1.15,2.5)	1.64(1.31,2.04)	1.45(1.22,1.73)	1.55(1.34,1.78)
1934-1938	1.33(1.21,1.45)	1.4(1,1.96)	1.4(1.15,1.71)	1.38(1.14,1.68)	1.28(1.15,1.43)	1.36(1.25,1.49)	1.43(1.19,1.7)	1.59(1.06,2.39)	1.53(1.03,2.28)	1.56(1.24,1.96)	1.33(1.11,1.6)	1.41(1.22,1.63)
1939-1943	1.21(1.1,1.33)	1.17(0.82,1.66)	1.3(1.05,1.6)	1.28(1.04,1.58)	1.18(1.05,1.32)	1.25(1.14,1.37)	1.29(1.07,1.56)	1.28(0.84,1.96)	1.38(0.91,2.11)	1.46(1.15,1.86)	1.2(0.99,1.46)	1.29(1.1,1.5)
1944-1948	1.05(0.95,1.17)	1.01(0.69,1.46)	1.13(0.9,1.42)	1.13(0.9,1.42)	1.08(0.95,1.23)	1.13(1.02,1.25)	1.14(0.93,1.39)	1.03(0.66,1.62)	1.22(0.78,1.91)	1.32(1.02,1.71)	1.11(0.9,1.37)	1.18(1,1.39)
1949-1953	0.97(0.86,1.08)	0.88(0.59,1.31)	1(0.79,1.28)	1.06(0.83,1.35)	0.98(0.86,1.12)	1.01(0.91,1.13)	1.06(0.85,1.31)	0.86(0.53,1.4)	1.12(0.7,1.81)	1.23(0.93,1.63)	1.01(0.8,1.27)	1.07(0.9,1.28)
1954-1958	0.91(0.81,1.03)	0.82(0.54,1.25)	0.95(0.73,1.23)	0.96(0.73,1.25)	0.91(0.79,1.06)	0.92(0.82,1.04)	0.99(0.78,1.25)	0.76(0.46,1.28)	1.05(0.63,1.76)	1.11(0.82,1.5)	0.93(0.73,1.2)	0.98(0.81,1.19)
1959-1963	0.83(0.72,0.94)	0.77(0.49,1.21)	0.83(0.62,1.11)	0.85(0.63,1.14)	0.87(0.74,1.02)	0.84(0.73,0.95)	0.87(0.67,1.13)	0.69(0.4,1.21)	0.9(0.52,1.57)	0.94(0.68,1.31)	0.88(0.67,1.15)	0.9(0.73,1.1)
1964-1968	0.75(0.65,0.87)	0.68(0.42,1.11)	0.71(0.52,0.98)	0.78(0.56,1.08)	0.84(0.7,1)	0.76(0.66,0.88)	0.8(0.6,1.06)	0.63(0.34,1.15)	0.79(0.43,1.44)	0.84(0.59,1.21)	0.83(0.62,1.12)	0.8(0.64,1)
1969-1973	0.68(0.58,0.8)	0.6(0.36,1.03)	0.62(0.44,0.88)	0.71(0.5,1.02)	0.75(0.62,0.91)	0.66(0.56,0.78)	0.72(0.53,0.99)	0.54(0.28,1.05)	0.69(0.36,1.34)	0.74(0.5,1.1)	0.75(0.54,1.04)	0.69(0.54,0.88)
1974-1978	0.61(0.51,0.74)	0.57(0.32,1.03)	0.55(0.37,0.82)	0.62(0.41,0.94)	0.69(0.55,0.85)	0.59(0.49,0.71)	0.61(0.42,0.87)	0.48(0.22,1.05)	0.57(0.26,1.22)	0.61(0.38,0.96)	0.63(0.43,0.92)	0.58(0.44,0.77)
1979-1983	0.56(0.45,0.7)	0.52(0.25,1.09)	0.51(0.32,0.83)	0.55(0.33,0.91)	0.63(0.49,0.83)	0.54(0.43,0.68)	0.53(0.34,0.84)	0.47(0.18,1.26)	0.47(0.17,1.28)	0.51(0.28,0.92)	0.59(0.38,0.93)	0.51(0.37,0.72)
1984-1988	0.54(0.39,0.74)	0.5(0.17,1.44)	0.51(0.26,1.02)	0.51(0.25,1.03)	0.59(0.42,0.85)	0.52(0.38,0.71)	0.44(0.22,0.89)	0.43(0.1,1.91)	0.39(0.08,1.89)	0.4(0.16,1.02)	0.5(0.25,1)	0.43(0.26,0.71)
1989-1993	0.53(0.3,0.92)	0.5(0.08,3.34)	0.53(0.16,1.77)	0.49(0.14,1.67)	0.53(0.28,1.02)	0.48(0.27,0.86)	0.37(0.11,1.24)	0.42(0.04,4.94)	0.33(0.02,4.67)	0.33(0.07,1.6)	0.41(0.12,1.43)	0.36(0.14,0.9)
1994-1998	0.45(0.08,2.55)	0.4(0,231.02)	0.46(0.01,20.29)	0.42(0.01,17.08)	0.45(0.06,3.53)	0.45(0.09,2.3)	0.29(0.01,8.02)	0.3(0,614.04)	0.24(0,406.16)	0.26(0,18.05)	0.34(0.01,10.12)	0.29(0.02,4.71)
